# Identifying nootropic drug targets via large-scale cognitive GWAS and transcriptomics

**DOI:** 10.1038/s41386-021-01023-4

**Published:** 2021-05-25

**Authors:** Max Lam, Chia-Yen Chen, Tian Ge, Yan Xia, David W. Hill, Joey W. Trampush, Jin Yu, Emma Knowles, Gail Davies, Eli A. Stahl, Laura Huckins, David C. Liewald, Srdjan Djurovic, Ingrid Melle, Andrea Christoforou, Ivar Reinvang, Pamela DeRosse, Astri J. Lundervold, Vidar M. Steen, Thomas Espeseth, Katri Räikkönen, Elisabeth Widen, Aarno Palotie, Johan G. Eriksson, Ina Giegling, Bettina Konte, Annette M. Hartmann, Panos Roussos, Stella Giakoumaki, Katherine E. Burdick, Antony Payton, William Ollier, Ornit Chiba-Falek, Deborah C. Koltai, Anna C. Need, Elizabeth T. Cirulli, Aristotle N. Voineskos, Nikos C. Stefanis, Dimitrios Avramopoulos, Alex Hatzimanolis, Nikolaos Smyrnis, Robert M. Bilder, Nelson B. Freimer, Tyrone D. Cannon, Edythe London, Russell A. Poldrack, Fred W. Sabb, Eliza Congdon, Emily Drabant Conley, Matthew A. Scult, Dwight Dickinson, Richard E. Straub, Gary Donohoe, Derek Morris, Aiden Corvin, Michael Gill, Ahmad R. Hariri, Daniel R. Weinberger, Neil Pendleton, Panos Bitsios, Dan Rujescu, Jari Lahti, Stephanie Le Hellard, Matthew C. Keller, Ole A. Andreassen, Ian J. Deary, David C. Glahn, Hailiang Huang, Chunyu Liu, Anil K. Malhotra, Todd Lencz

**Affiliations:** 1 Division of Psychiatry Research, The Zucker Hillside Hospital, Glen Oaks, NY USA; 2 Stanley Center for Psychiatric Research, Broad Institute of Harvard and MIT, Cambridge, MA USA; 3 Analytic and Translational Genetics Unit, Massachusetts General Hospital, Boston, MA USA; 4 Institute for Behavioral Science, Feinstein Institutes for Medical Research, Manhasset, NY USA; 5 Institute of Mental Health, Singapore, Singapore; 6 Biogen, Inc, Cambridge, MA USA; 7 Psychiatric and Neurodevelopmental Genetics Unit, Massachusetts General Hospital, Boston, MA USA; 8 Center for Medical Genetics & Hunan Key Laboratory of Medical Genetics, School of Life Sciences, Central South University, Changsha, China; 9 Psychiatry Department, SUNY Upstate Medical University, Syracuse, NY USA; 10 Lothian Birth Cohorts, University of Edinburgh, Edinburgh, Scotland UK; 11 Lothian Birth Cohorts group, Department of Psychology, University of Edinburgh, Edinburgh, UK; 12 Department of Psychiatry and the Behavioral Sciences, Keck School of Medicine, University of Southern California, Los Angeles, CA USA; 13 Tommy Fuss Center for Neuropsychiatric Disease Research, Boston Children’s Hospital, Boston, MA USA; 14 Department of Psychiatry, Harvard Medical School, Boston, MA USA; 15 Olin Neuropsychic Research Center, Institute of Living, Hartford Hospital, Hartford, CT USA; 16 Regeneron Pharmaceuticals, Inc., Tarrytown, NY USA; 17 Department of Psychiatry, Icahn School of Medicine at Mount Sinai, New York, NY USA; 18 Department of Genetics and Genomic Science and Institute for Multiscale Biology, Icahn School of Medicine at Mount Sinai, New York, NY USA; 19 Department of Medical Genetics, Oslo University Hospital, Oslo, Norway; 20 NORMENT, Department of Clinical Science, University of Bergen, Bergen, Norway; 21 Division of Mental Health and Addiction, Oslo University Hospital, Oslo, Norway; 22 Spaulding Rehabilitation Hospital Boston, Charlestown, MA USA; 23 Dr. Einar Martens Research Group for Biological Psychiatry, Department of Medical Genetics, Haukeland University Hospital, Bergen, Norway; 24 Department of Psychology, University of Oslo, Oslo, Norway; 25 Department of Psychiatry, Zucker School of Medicine at Hofstra/Northwell, Hempstead, NY USA; 26 Department of Biological and Medical Psychology, University of Bergen, Bergen, Norway; 27 Department of Psychology and Logopedics, Faculty of Medicine, University of Helsinki, Helsinki, Finland; 28 Institute for Molecular Medicine Finland (FIMM), University of Helsinki, Helsinki, Finland; 29 Wellcome Trust Sanger Institute, Wellcome Trust Genome Campus, Cambridge, UK; 30 Department of Medical Genetics, University of Helsinki and University Central Hospital, Helsinki, Finland; 31 Department of General Practice, University of Helsinki and Helsinki University Hospital, Helsinki, Finland; 32 Department of Obstetrics & Gynaecology, Yong Loo Lin School of Medicine, National University of Singapore, Singapore, Singapore; 33 Folkhälsan Research Center, Helsinki, Finland; 34 Department of Psychiatry, Martin Luther University of Halle-Wittenberg, Halle, Germany; 35 Mental Illness Research, Education, and Clinical Center (VISN 2), James J. Peters VA Medical Center, Bronx, NY USA; 36 Department of Psychology, University of Crete, Crete, Greece; 37 Department of Psychiatry - Brigham and Women’s Hospital, Harvard Medical School, Boston, MA USA; 38 Division of Informatics, Imaging & Data Sciences, School of Health Sciences, The University of Manchester, Manchester, UK; 39 Centre for Epidemiology, Division of Population Health, Health Services Research & Primary Care, The University of Manchester, Manchester, UK; 40 School of Healthcare Sciences, Manchester Metropolitan University, Manchester, United Kingdom; 41 Division of Translational Brain Sciences, Department of Neurology, Bryan Alzheimer’s Disease Research Center, and Center for Genomic and Computational Biology, Duke University Medical Center, Durham, NC USA; 42 Psychiatry and Behavioral Sciences, Division of Medical Psychology, and Department of Neurology, Duke University Medical Center, Durham, NC USA; 43 William Harvey Research Institute, Queen Mary University of London, London, UK; 44 Helix Inc., San Diego, CA USA; 45 Campbell Family Mental Health Institute, Centre for Addiction and Mental Health, University of Toronto, Toronto, ON Canada; 46 2nd Department of Psychiatry, National and Kapodistrian University of Athens Medical School, University General Hospital “ATTIKON”, Athens, Greece; 47 University Mental Health Research Institute, Athens, Greece; 48 Neurobiology Research Institute, Theodor-Theohari Cozzika Foundation, Athens, Greece; 49 Department of Psychiatry, Johns Hopkins University School of Medicine, Baltimore, MD USA; 50 Department of Genetic Medicine, Johns Hopkins University School of Medicine, Baltimore, MD USA; 51 UCLA Semel Institute for Neuroscience and Human Behavior, Los Angeles, CA USA; 52 Department of Psychiatry, Yale University School of Medicine, New Haven, CT USA; 53 Department of Psychology, Yale University, New Haven, CT USA; 54 Department of Psychology, Stanford University, Palo Alto, CA USA; 55 Robert and Beverly Lewis Center for Neuroimaging, University of Oregon, Eugene, OR USA; 56 23andMe, Inc., Mountain View, CA USA; 57 Weill Cornell Psychiatry at NewYork-Presbyterian, Weill Cornell Medical Center, New York, NY USA; 58 Laboratory of NeuroGenetics, Department of Psychology & Neuroscience, Duke University, Durham, NC USA; 59 Clinical and Translational Neuroscience Branch, Intramural Research Program, National Institute of Mental Health, National Institute of Health, Bethesda, MD USA; 60 Lieber Institute for Brain Development, Johns Hopkins University Medical Campus, Baltimore, MD USA; 61 Neuroimaging, Cognition & Genomics (NICOG) Centre, School of Psychology and Discipline of Biochemistry, National University of Ireland, Galway, Ireland; 62 Neuropsychiatric Genetics Research Group, Department of Psychiatry and Trinity College Institute of Neuroscience, Trinity College Dublin, Dublin, Ireland; 63 Division of Neuroscience and Experimental Psychology/School of Biological Sciences, Faculty of Biology Medicine and Health, Manchester Academic Health Science Centre, Salford Royal NHS Foundation Trust, University of Manchester, Manchester, UK; 64 Department of Psychiatry and Behavioral Sciences, Faculty of Medicine, University of Crete, Heraklion, Crete, GR Greece; 65 Helsinki Collegium for Advanced Studies, University of Helsinki, Helsinki, Finland; 66 Institute for Behavioral Genetics, University of Colorado, Boulder, CO USA; 67 Institute of Clinical Medicine, University of Oslo, Oslo, Norway

**Keywords:** Heritable quantitative trait, Genetics research

## Abstract

Broad-based cognitive deficits are an enduring and disabling symptom for many patients with severe mental illness, and these impairments are inadequately addressed by current medications. While novel drug targets for schizophrenia and depression have emerged from recent large-scale genome-wide association studies (GWAS) of these psychiatric disorders, GWAS of general cognitive ability can suggest potential targets for nootropic drug repurposing. Here, we (1) meta-analyze results from two recent cognitive GWAS to further enhance power for locus discovery; (2) employ several complementary transcriptomic methods to identify genes in these loci that are credibly associated with cognition; and (3) further annotate the resulting genes using multiple chemoinformatic databases to identify “druggable” targets. Using our meta-analytic data set (*N* = 373,617), we identified 241 independent cognition-associated loci (29 novel), and 76 genes were identified by 2 or more methods of gene identification. Actin and chromatin binding gene sets were identified as novel pathways that could be targeted via drug repurposing. Leveraging our transcriptomic and chemoinformatic databases, we identified 16 putative genes targeted by existing drugs potentially available for cognitive repurposing.

## Introduction

One central goal for genome-wide association studies (GWAS) is the identification of potential targets for clinically useful pharmacologic interventions; drugs whose targets have supporting genetic evidence of association to the indication are significantly more likely to successfully reach approval than those without such evidence [1]. While novel drug targets for major psychiatric illnesses have emerged from recent large-scale GWAS [2–4], broad-based cognitive deficits are an enduring and disabling feature for many patients with severe mental illness and are inadequately addressed by current medications [5]. Similarly, effective cognitive enhancing medications (“nootropics”) are limited for patients with dementias and other neurodegenerative disorders [6]. Thus, the genetic study of general cognitive ability (GCA) holds the potential for identifying novel targets for nootropic medications, which could have widespread applications [7].

The genetic architecture of GCA has been examined with increasingly large sample sizes over the last few years [8–10]. Physical health, illness, mortality [11], and psychiatric traits [12] have shown significant genetic correlations with individual differences in GCA. Dissecting the pleiotropic genetic architectures underlying GCA, educational attainment, and schizophrenia, we have recently shown that neurodevelopmental pathways and adulthood synaptic processes are dissociable etiologic mechanisms relating to genetic liability to psychosis [13].

Nevertheless, identifying specific genes functionally linked to GCA, with protein products that could be targeted by pharmacological agents, remains a core challenge. Using a pathway-based methodology [14], we previously reported that several genes encoding T- and L-type calcium channels, targeted by known pharmaceuticals, were associated with GCA [7]; however, that study was relatively underpowered. Now with much larger GWAS of cognition available [8, 9], increasingly large regions of the genome may demonstrate statistical association with GCA, requiring a principled approach to identify treatment-relevant genes within those regions. Fortunately, the recent release of large-scale brain eQTL/transcriptomic databases have substantially enhanced the assignment of regional GWAS signals to specific genes [15–20]. Simultaneously, recent advances in genetic epidemiology methods (e.g. Mendelian randomization) have enabled identification of potentially spurious eQTL associations that may be based on linkage rather than meaningful biology [21]. Thus, the convergence of adequately powered samples coupled with cutting-edge statistical and bioinformatics tools allows for novel genetic mechanisms underlying GCA to be discovered.

In order to turn the resulting GCA-associated gene sets into actionable nootropic drug targets, it is useful to limit the search to those genes which encode proteins that are known to be druggable [22]. However, novel technologies have nominated an increasing number of potentially druggable genes [23–25] beyond the 15% of the genome originally estimated based on fundamental pharmacologic principle*s* [22]. Given this uncertainty, well-characterized existing compounds present the most robust evidence of demonstrable druggability for a given target, suggesting the efficiency of drug repurposing/repositioning as a primary research strategy [26]. Such drug repurposing efforts also serve to reduce the high rate of failure associated with novel drug discovery [27]. Applied to GWAS of psychiatric disorders, drug repurposing studies have pointed towards glutamatergic modulators and calcium channel blockers in schizophrenia and sex hormones in depression; such analyses consistently also point towards existing antipsychotic and antidepressant compounds, serving as a positive control for the drug repurposing approach [2, 4, 28, 29].

Here, we jointly analyzed the two largest GWAS of cognition to date [8, 9]. In doing so, we harmonized the genome-wide signals associated with GCA across these studies at the levels of both individual variants and broader genomic regions of loci and pathways. We also employed novel analytical methods not previously employed in cognitive GWAS studies to determine the direction of causality between GWAS hits for GCA and genetically correlated phenotypes. Large brain-based transcriptomic databases were then utilized to determine the biological underpinnings of the most credible and actionable cognitive GWAS signals. Finally, we sought converging evidence from large-scale chemoinformatic resources to identify the most promising nootropic drug targets for drug repurposing.

## Materials and methods

**Fig. 1:**
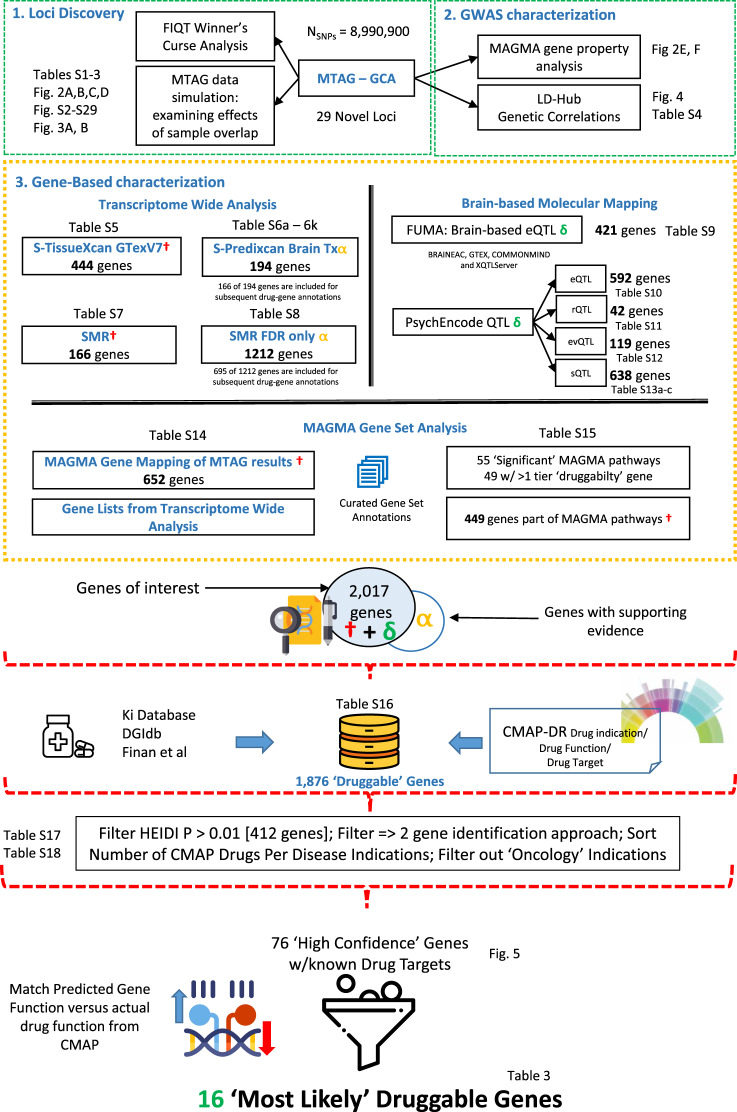
Workflow for the present study. The overall data analytic strategy follows a broad strategy of (i) locus discovery, (ii) gene-based characterization, and (iii) gene-to-drug annotations. The green box at top summarizes locus discovery procedures and characterization of results. Yellow box summarizes downstream analysis of summary statistics, resulting in a set of genes available for druggability analysis displayed at figure bottom (red brackets). At each step, location of further details in Tables, Figures, and Supplementary Materials is specified. SMR summary statistics mendelian randomization, FUMA functional mapping and annotation of GWAS, eQTL expression quantitative trait locus, rQTL ribosomal occupancy qtl, sQTL splicing qtl, evQTL expression variation qtl.

A broad study overview is provided in Fig. 1 and specific algorithm or software carried out in each analysis stage provided in Table S1, respectively. Further in-depth details are available in the ‘Materials and Methods’ section within the Supplementary Materials. The overall data analytic strategy follows a broad strategy of (i) Locus discovery (ii) Gene-based characterization (iii) Gene-to-drug annotations.

### Locus discovery

The core analysis combined summary statistics of the two largest GWAS of cognition to date [8, 9]. Savage et al (*N* = 269,867) analyzed 9,395,118 single-nucleotide polymorphisms (SNPs) for association to intelligence, and Davies et al. [9] (*N* = 283,531) analyzed 12,871,771 SNPs in relation to the somewhat broader general cognition phenotype. The latter set of summary statistics reported by Davies et al. [9] was reduced from the original *N* = 300,486 due to data access limitations. First, we carried out MTAG as meta-analysis. As in all genome-wide meta-analytic studies, we expected to gain and lose some loci. Hence, *Winner’s Curse Adjustment* via the FDR Inverse Quantile Transformation was carried out to evaluate if loci no longer significant were in fact due to winner’s curse. The two studies were noted to have a relatively large degree of sample overlap (89%); the relative increase in power available is more modest than would be expected for a meta-analysis of fully independent samples. While statistical inflation of the combined results is thought to be well-controlled by MTAG [30], the larger than usual sample overlap was not addressed by the original MTAG report. We therefore carried out data simulation in scenarios of 75 and 88.9% sample overlap to extend current understanding of how MTAG might appropriately handle the effects of sample overlap that is relevant to the results reported in the current study. Genome-wide GCA association results were passed through the FUMA v1.3.5 pipeline [31] (see Supplementary Materials: Materials and Methods) to identify GWAS-significant independent SNPs and genomic loci and perform other downstream analyses that we report in subsequent sections. The genome-wide significant threshold was set at *P* < 5e−8. MTAG results were characterized using genetic correlations with other psychiatric and physical traits via LD-hub [32], and gene property analysis [33] was performed to screen gene expression and localization in CNS tissue. Although many of the results from genetic correlation and gene expression screening are expected to be reported in prior cognitive GWAS [8, 9], they are conducted as “sanity check” to the genome-wide GCA results that are reported here.

### Gene-based characterization

In the second phase of the analysis, we utilized a series of approaches to identify potential genes emanating from the MTAG genome-wide results. These ranged from targeted transcriptome wide approaches, to co-located gene expression eQTL mapping, to more general gene-set and pathway-based methods. The objective is to elucidate a broadly inclusive list of genes associated with GCA that could be subjected to downstream gene-drug annotations. First, S-PrediXcan and S-TissueXcan [34] Transcriptome-Wide Analysis (TWAS) were used to characterize gene expression mediated associations in GTEx(v7) tissues. The latter was used to identify potentially functional genes in general, and the former was used to identify genes that are expressed specifically in the brain. We also carried out Summary Statistics Based Mendelian Randomization (SMR) and Heterogeneity in Dependent Instruments (HEIDI) analyses [21] where SNPs are used as instruments to identify gene expression effects on a GCA with estimated SNP-gene expression and SNP-phenotype effects entered into a formal mediation model. At the same time, the HEIDI test identifies SNP-gene expression effects and SNP-phenotype effects that are correlated with each other through LD rather than biologically related via horizontal or vertical pleiotropy. SMR/HEIDI allows us to prioritize genes that might have an immediate relationship between its function and phenotype variance and de-prioritize genes that tend to be in regions with long range LD, which might require further experimental follow up to clarify their function. Next, we carried out molecular quantitative trait locus mapping to identify genomic regions where association signals and gene expression “hot spots” in brain tissue tend to be co-located. Annotation databases that are part of the FUMA pipeline were utilized for this step (See Supplementary Materials: Materials and Methods). Additional molecular annotations from the PsychENCODE database such as splicing variants, expression variation QTL, and ribosomal occupancy QTL were also examined. Gene association tests and gene set analysis were carried out via MAGMA [33]. Results of gene-based tests from MTAG genome-wide results (via MAGMA gene mapping), SMR, and S-TissueXcan were included in gene-set analysis. Genes surviving multiple testing correction in the gene-mapping analysis and nominally significant genes that were part of significant gene sets were selected for downstream drug-gene annotations.

### Gene-drug annotations

“Druggable” genes from the Drug-Gene Interaction database (DGIdb v.2), Psychoactive Drug Screening Database K_i_DB, and a recent review on “druggability” [35] were consolidated. Genes that were identified by transcriptome wide analysis, molecular mapping QTL, and gene-set analysis were filtered on the consolidated druggable gene list from drug databases. At the final stage of the analysis we annotated high confidence genes using the Broad Institute Connectivity Map, Drug Re-purposing Database [36] that provides more in-depth details such as drug names, mechanism of action, and drug indicatons. We matched drug mechanisms of action (MOA) with direction of effects obtained from earlier transcriptome analyses. Genes that are filtered in this manner are deemed as high likelihood for being potential therapeutic targets for nootropic repurposing.

## Results

We have prepared a study flowchart (see Fig. 1) as well as a methodological overview and results roadmap (see Table S1) to aid in navigating through the reported results.

### Locus discovery: MTAG of general cognitive ability

A total of 8,990,900 SNPs present in both sets of summary statistics were extracted for use in the MTAG meta-analysis of the two largest GWAS of cognition [8, 9]. Since both sets of GWAS summary statistics indexed GCA, we constrained MTAG analysis to give a single output; MTAG was further constrained such that the heritability of both sets of summary statistics were set to equivalent and genetic covariance set to 1 – an approach not unlike fixed effect meta-analysis. Potential inflationary effects of sample overlap, as tested by simulations in scenarios of 75 and 88.9% sample overlap, showed no inflation in either scenario (*β*_75%_ = 1.011; *β*_88.9%_ = 1.015) and good agreement between GWAS of the full sample, and MTAG of the GWAS of subsamples with substantial sample overlap (Adj. *R*^2^_75%_ = 0.94; Adj. *R*^2^_88.9%_ = 0.96; see Fig. S1). MTAG’s *maxFDR* analysis revealed low probability of false positives (*max*_*fdr*_ = 4.5e–7). The resulting mean chi-square values after carrying out MTAG were as follows: mean χ^2^_Savage_ = 1.624, mean χ^2^_Davies_ = 1.544, and mean χ^2^_*MTAG*_ = 1.783. The average projected GWAS equivalent sample size after MTAG analysis was 373,617, which shows substantial power improvement over input GWAS.

**Fig. 2:**
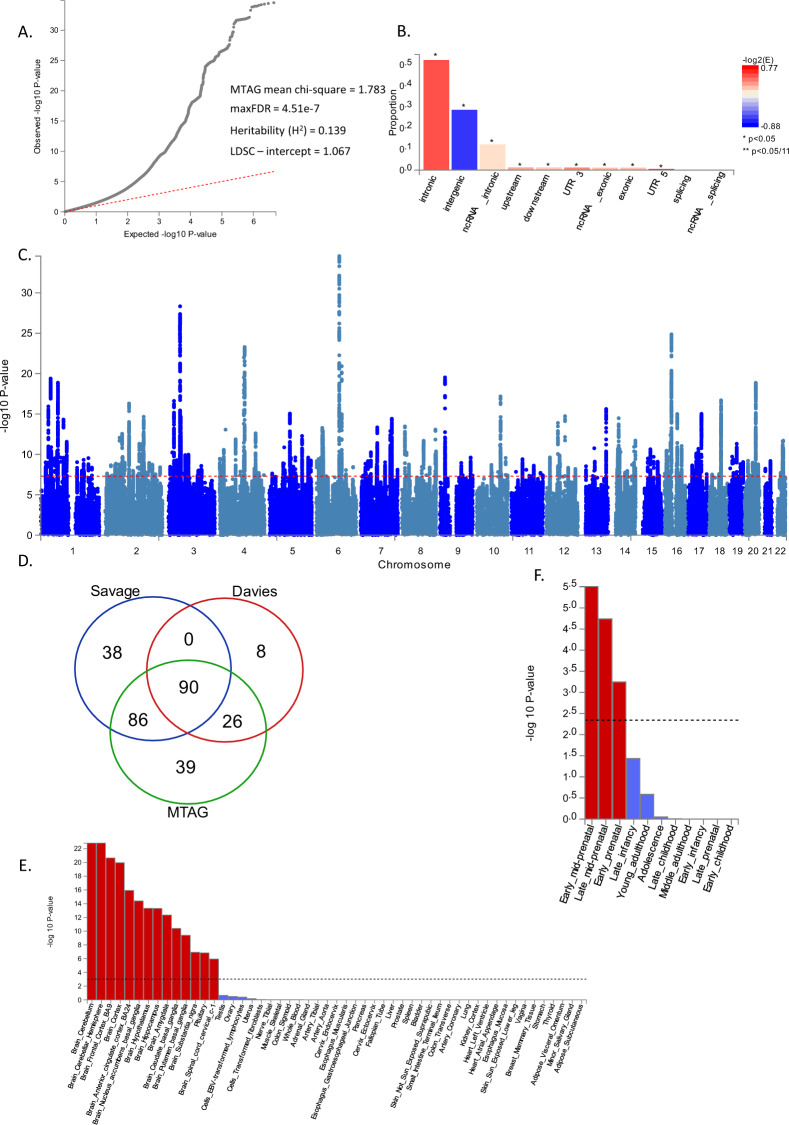
GWAS association plots for Cognitive MTAG. **A** QQ-plot. **B** SNP annotation plot. **C** Manhattan plot for General Cognitive Ability. **D** Venn Diagram showing loci overlap for input GWAS and MTAG results. **E** MAGMA gene property analysis for overall GTEXv7. **F** MAGMA gene property analysis using BrainSpan.

Clumping procedures were carried out on 8,990,900 SNPs (see Methods). Genome-wide significant loci were defined based on R^2^ > 0.6, and loci within 250 kb of each other were merged. A total of 241 loci were GWAS significant for the MTAG analysis (Fig. 2A–D), while 214 loci and 124 loci were GWAS significant for Savage et al. [8] and Davies et al. [9] respectively (aee Table S2). It should be noted that 38 loci reported as significant in Savage et al. [8] and 8 loci in Davies et al. [9] were no longer significant in the MTAG analysis (Table S3). Winner’s curse analysis suggested that these loci were likely false positives in the original studies (Table S4). A total of 39 MTAG-significant loci were not reported as significant in the input GWAS (Table S3). We then looked up reports that have used multi-trait strategies to enhance power for GCA [10, 13] and found that of the 39 loci, 8 loci were also reported by Hill et al. [10], 1 locus was reported by Lam et al. [13], and 1 locus was reported by both of these studies (Fig S2). Region plots of fully novel loci are reported in Figs. S3–S30.

### Genome-wide characterization

A preliminary screen of the genome-wide MTAG via the MAGMA gene-property analysis confirms that the genetic architecture of GCA is closely related to gene expression within various brain tissues after Bonferroni correction (Fig. 2E, F). At the same time, we observe that GCA is also related to genes expressed in prenatal brain tissue implicating a role for early neurodevelopment.

Genetic correlations were estimated between GCA and 855 phenotypes from LD-hub [32] and UK Biobank. MTAG summary statistics were merged and aligned with HapMap3 SNPs excluding the MHC region for genetic correlation analysis (1,190,946 SNPs remained). In total, 297 phenotypes showed significant genetic correlation with cognition at Bonferroni corrected *P* < 0.05. Consistent with prior reports [7, 10, 37, 38], traits genetically correlated with GCA included education, reproduction, longevity, personality, smoking behavior, anthropometric, brain volume, psychiatric, dementias, lung function, sleep, glycemic, autoimmune, cardio-metabolic, cancer and several ICD-10 medical phenotypes (Table S5). Several novel traits that have not been previously reported to be genetically correlated with GCA are displayed in Fig S3; these include several gastric, vascular, and bronchial diagnoses and medications.

### Gene-based characterization

In the next several sections, we report results of a series of analyses that aimed to nominate genes associated with GCA via a series of transcriptomic and pathway-based approaches applied to SNP-based summary statistics obtained in the earlier MTAG analysis. As described (see Fig.1; Table S1, “Gene-Based Characterization”), a variety of complementary transcriptomic analysis were conducted to convert SNP/locus associations into directional, biologically interpretable gene effects on GCA. In the sections below, we take an inclusive approach to generate a broad list of candidate genes which will be subjected to further downstream gene-drug annotations. Moreover, by utilizing a range of complementary functional characterization approaches, the resulting gene list can be prioritized in terms of total strength of supporting evidence (which will be summarized in Table S18).

### S-PrediXcan/S-TissueXcan

Genome-wide joint transcriptomic modeling carried out via S-TissueXcan [34] analysis in all 48 GTExv7 tissues yielded 444 significant genes after Bonferroni correction (Table S6). We also conducted brain tissue-specific transcriptome wide modeling via S-PrediXcan and found that 194 genes were significant in one or more brain tissue annotations (Table S7a–k).

### SMR and HEIDI

Using Summary-stats-based Mendelian Randomization (SMR [21]), we were able to identify 166 genes that were genome-wide significant (Bonferroni corrected), whose gene expression levels were contributing to variation of GCA (Table S8). As discussed in the Methods and Materials section (Supplementary Materials), SMR analysis tends to be more conservative than other gene identification methodologies [34]. Hence, we also nominated 1212 genes nominally significant genes (*P*_SMR_ < 0.05) for follow up in the later gene annotation (Table S9). Importantly, SMR analysis indicated that the colocalization of GWAS and eQTL signals in 412 genes was likely artifactual due to linkage disequilibrium, rather than causal mediation, as indicated by *P*_*HEIDI*_ < 0.01. These were excluded from subsequent druggability analysis.

### Brain-based molecular QTL mapping

Next, we utilized eQTL mapping approaches to identify expressed genes in and around genome-wide significant regions within the MTAG results. We leveraged on databases packaged with FUMA [31] such as BRAINEAC, GTEx, COMMONMIND and XQTLServer for initial eQTL mapping. eQTL mapping from the FUMA [31] pipeline revealed 421 significantly expressed genes within GWAS significant regions (FDR corrected *p* values; Table S10). Additional molecular QTL mapping of PsychENCODE prefrontal cortex with GCA SNPs identified 592 genes with eQTL (Table S11), 42 genes implicated in ribosomal occupancy QTLs (Table S12), 119 genes with expression variation QTLs (Table S13) and 638 genes with splicing activity QTLs (Tables S7a–S14c).

### MAGMA association tests and gene set analysis

MAGMA gene-based analysis revealed that 652 of 18,730 genes were significantly associated with GCA after Bonferroni correction (Table S15). MAGMA gene-set analyses were carried out using gene lists derived from the MAGMA gene-based results, as well as from lists of genes significant in the SMR analysis (only using PsychENCODE results), and S-TissueXcan (Using GTExv7) output. Full gene-set analysis results are presented in Table S16.

Gene set results derived from the MAGMA gene-based results were highly consistent with findings of previous cognitive GWAS: gene sets that have been associated with neuropsychiatric disorders such as schizophrenia and ASD were highly significant, congruent with significant genetic correlations between GCA and these disorders. Relatedly, gene sets reflecting neurodevelopmental processes implicated in schizophrenia and ASD, including the CHD8, FMRP, and RBFOX pathways, were also implicated in GCA [39]. Also consistent with prior reports, a series of neuronal and dendritic development, differentiation, and regulation gene sets were also associated with GCA [10].

There were also several classes of gene sets emerging from our data that are novel with respect to GCA; notably, these results emerged in the context of the SMR (Table 1a) and S-TissueXcan (Table 1b) results, demonstrating the value of leveraging multiple approaches to post-GWAS gene identification. First, genes responsible for cellular response to small molecules such as sugars and cytokines appear to be implicated. Cell signal transductions mediated by small monomeric GTPases also appear to be relevant for GCA. In addition, gene sets underpinning cell structure and binding mechanisms, including adhesion, protein complexes, actin and chromatin binding were identified.

**Table 1:** (a) SMR and (b) S-TissueXcan gene sets associated with cognitive function. Only novel results are displayed.

Gene sets	Gene Set P	Gene set categories
(a) SMR		
*GOBP:regulation_of_binding*	8.54E−06	Cell binding
*REACTOME:Signaling_by_Rho_GTPases*	2.28E−06	Cell metabolism
*GOBP:small_GTPase_mediated_signal_transduction*	2.13E−05	Cell metabolism
*REACTOME:Signaling_by_Rho_GTPases*	3.05E−07	Cell metabolism
*GOBP:nucleosome_organization*	5.92E−06	Cell structure
*GOBP:protein-DNA_complex_assembly*	1.81E−06	Cell structure
*GOBP:protein-DNA_complex_subunit_organization*	2.17E−07	Cell structure
*GOBP:cell-substrate_adhesion*	5.06E−06	Cell structure
*GOBP:response_to_glucose*	4.80E−06	Interaction with small molecules
*GOBP:response_to_hexose*	3.73E−06	Interaction with small molecules
*GOBP:response_to_monosaccharide*	8.65E−06	Interaction with small molecules
*GOBP:macromolecule_methylation*	4.62E−08	Methylation
*GOBP:methylation*	3.29E−07	Methylation
*REACTOME:Ion_channel_transport*	1.00E−05	Neuronal/Dendritic regulation/development

(b) S-TissueXcan		
*GOMF:actin_binding*	6.44E−06	Cell binding
*GOMF:chromatin_binding*	4.52E−06	Cell binding
*GOBP:cellular_macromolecular_complex_assembly*	1.71E−07	Cell structure
*GOBP:cellular_protein_complex_assembly*	1.68E−07	Cell structure
*GOBP:cellular_response_to_interferon-gamma*	2.67E−06	Interaction with small molecules

Given the strong signals derived from gene-set analysis, we extracted all nominally significant genes (*P* < 0.05) within each significant MAGMA gene set for further downstream annotations. A total of 449 genes were identified as part of significant MAGMA pathways (Table S16), and were subjected to further chemoinformatic analysis for druggability as described below.

### Identifying drug-gene targets for nootropic re-purposing

A total of 2017 genes (see Fig. 1) were identified via the gene characterization approaches described above. To consolidate evidence for these 2017 nominated genes, we performed the following steps: First, we combined genes identified by MAGMA gene-set and gene-mapping analysis, genes identified via eQTL methods, and genes identified via transcriptome wide analysis (Bonferroni corrected). 695 SMR identified genes, 166 genes identified by S-PrediXcan brain tissue eQTL analysis were added to the annotation. 1876 “druggable” genes were identified by merging across several drug databases (see Supplementary Materials: Materials and Methods, Table S17). After applying a preliminary filter based on three requirements (druggable genes, genes with *P*_HEID*I*_ > 0.01, and genes that were identified by two or more gene-identification approaches), 91 genes remained. It should be noted that gene sets representing methylation processes, DNA complex, and nucleosomes, while significantly associated with GCA, do not contain any genes that are targeted by known drugs, based upon our druggability criteria described in the Materials and Methods section.

**Fig. 3:**
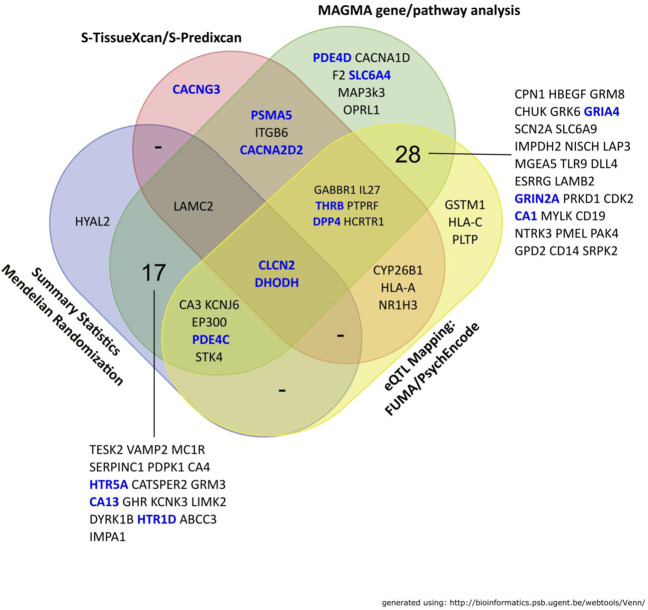
Venn diagram of “High Confidence” genes and gene identification approaches. Genes highlighted in blue were deemed as most likely having gene targets that were suitable for nootropic re-purposing.

We further annotated these genes with information from the Broad Institute CMAP Drug Repurposing Database [36]. We filtered out drug indications for “Oncology” mainly for drug delivery concerns, yielding a final list of 76 “high-confidence” genes that were deemed druggable (Fig. 3). According to the Cell-type Specific Expression Analysis (CSEA) [40], these genes are expressed in a broad range of CNS cell types, including cortical, subcortical, and cerebellar neurons, as well as astrocytes and oligodendrocytes (Table S20); moreover, expression of these genes is broadly demonstrated across developmental epochs (Table S21). These genes were annotated with eQTL directions (i.e., up- or down-regulation associated with higher GCA) for each gene; eQTL directions were obtained from earlier analysis, including brain-eQTLs from S-PrediXcan, SMR, PsychENCODE eQTL, RNA-seq Ribosomal and Splicing eQTL mapping, and overall S-TissueXcan GTEX eQTL analysis (Table S18). Effect sizes that indicated up-regulation of the gene associated with higher GCA were denoted as “↑,” while those where down-regulation was associated with higher GCA were denoted as “↓.” We predicted the “mechanism of action” from the overall eQTL direction to determine if a given gene might require either an “Agonist” or “Antagonist” to enhance GCA. This was achieved by taking the sum of eQTL directions across tissues (see Table S18). If overall eQTL indicates up-regulation, it would more likely require an agonist and vice-versa. We eliminated “Ambiguous” gene targets that have an equal number of tissues that show up- and down-regulated gene expression.

Annotations from the CMAP Drug Re-purposing Database, which include drug names, mechanism of action (MOA), and drug indications, were merged with these “high-confidence” genes (Table S19). Of the 76 “high-confidence” genes, manual curation of the CMAP annotations compared to the predicted mechanism of action based on eQTL directions yielded a final set of 16 most likely targets for drug repurposing; these potential drugs and their current indications with various types of physical or psychiatric conditions are listed in Table 2. Notably, the relationships between some of the drug MOA and gene targets do not always appear to be direct. For example, adrenergic receptor agonists can indirectly activate calcium channels of which *CACNA2D2* is a constituent.

**Table 2:** Prioritized genes for nootropic re-purposing.

Gene ID	Gene name	Predicted nootropic function	Drug name(s)	MOA	Drug indications
*CA1*	Carbonic anhydrase 1	Antagonist	**Acetazolamide** **Benzthiazide** **Brinzolamide** **Chlorthalidone** **Diclofenamide** **Dorzolamide** **Ethoxzolamide** **Methazolamide** ***Topiramate*** *Trichlormethiazide* *Methyclothiazide* *Levosulpiride* *Zonisamide* *Bendroflumethiazide* *Hydroflumethiazide* *Coumarin* *Amlodipine*	• **Carbonic anhydrase inhibitor** • *Glutamate receptor antagonist* • *Kainate receptor antagonist* • *Chloride channel blocker* • *Chloride reabsorption inhibitor* • *Dopamine receptor antagonist* • *Sodium channel blocker* • *T-type calcium channel blocker* • *Sodium/potassium/chloride transporter inhibitor* • *Vitamin K antagonist* • *Calcium channel blocker*	**Congestive heart failure** **Duodenal ulcer disease** **Dyspepsia** **Edema** **Epilepsy** **Glaucoma** **Hypertension** **Migraine headache** **Ocular hypertension** *Acute glomerulonephritis (AGN)* *Anxiety* *Asthma* *Celiac disease* *Chronic renal failure* *Chronic stable angina* *Coronary artery disease (CAD)* *Hepatic cirrhosis* *Irritable bowel syndrome* *Nephrotic syndrome* *Premature ejaculation (PE)* *Psychosis* *Schizophrenia* *Seizures* *Ulcerative colitis* *Vertigo*
*CA13*	Carbonic anhydrase 13	Antagonist	**Ethoxzolamide** *Zonisamide*	• **Carbonic anhydrase inhibitor** *• Sodium channel blocker* *• T-type calcium channel blocker*	**Glaucoma** **Duodenal ulcer disease** *Epilepsy*
*CACNA2D2*	Calcium voltage-gated channel auxiliary subunit alpha2delta 2	Agonist	*Gabapentin-Enacarbil*	*Adrenergic receptor agonist*	*Restless leg syndrome* *Postherpetic neuralgia*
*CACNG3*	Calcium voltage-gated channel auxiliary subunit gamma3	Agonist	*Gabapentin-enacarbil*	*Adrenergic receptor agonist*	*Restless leg syndrome* *Postherpetic neuralgia*
*CLCN2*	Chloride voltage-gated channel 2	Agonist	**Lubiprostone**	**Chloride channel activator**	**Constipation** **Irritable bowel syndrome**
*DHODH*	Dihydroorotate dehydrogenase (quinone)	Antagonist	***Leflunomide*** *Atovaquone* *Teriflunomide*	• **Dihydroorotate dehydrogenase inhibitor** • *Mitochondrial electron transport inhibitor* • *PDGFR tyrosine kinase receptor inhibitor*	**Multiple Sclerosis** **Rheumatoid Arthritis** *Pneumonia*

*DPP4*	Dipeptidyl peptidase 4	Antagonist	**Alogliptin** **Anagliptin** **Linagliptin** **Saxagliptin** **Sitagliptin** **Teneligliptin** **Trelagliptin** **Vildagliptin** *Atorvastatin*	• **Dipeptidyl** **peptidase inhibitor** • *HMGCR inhibitor*	**Diabetes mellitus** **stroke** *Cholesterol reduction*
*GRIA4*	Glutamate ionotropic receptor AMPA type subunit 4	Agonist	*Piracetam*	*Acetylcholine agonist*	*Senile dementia*
*GRIN2A*	Glutamate ionotropic receptor NMDA type subunit 2 A	Antagonist	**Acamprosate** **Amantadine** **Telbamate** **Halothane** **Memantine** *Atomoxetine* *Milnacipran* *Gabapentin*	• Glutamate **receptor antagonist** • *Norepinephrine transporter inhibitor* • *Serotonin-norepinephrine reuptake inhibitor (SNRI)* • *Calcium channel blocker*	**Abstinence from alcohol** **Alzheimer’s disease** **Epilepsy** **General anaesthetic** **Influenza A virus infection** **Parkinson’s Disease** **Restless leg syndrome** **Senile dementia** **Virus herpes simplex (HSV)** *Attention deficit/hyperactivity disorder (ADHD)* *Fibromyalgia* *Seizures* *Pain from shingles*
*HTR1D*	5-hydroxytryptamine receptor 1D	Agonist	**Serotonin** **Almotriptan** **Dihydroergotamine** **Eletriptan** **Frovatriptan** **Naratriptan** **Rizatriptan** **Sumatriptan** **Zolmitriptan** **Aripiprazole** *Oxymetazoline* *Bromocriptine* *Cabergoline* *lIsuride* *Pramipexole* *Ropinirole*	• **Serotonin receptor agonist** • *Adrenergic receptor agonist* • *Dopamine receptor agonist* • *Growth factor receptor activator*	**Bipolar disorder** **Depression** **Migraine headache** **Schizophrenia** **Sleeplessness** *Acromegaly* *Hyperprolactinemia* *Nasal congestion* *Parkinson’s Disease* *Restless leg syndrome*

*HTR5A*	5-hydroxytryptamine receptor 5 A	Antagonist	**Ergotamine** **Yohimbine** **Asenapine** **Clozapine** **Loxapine** **Olanzapine** **Vortioxetine** **Ketanserin** **Methysergide**	• Serotonin **receptor antagonist** • *Adrenergic receptor antagonist* • *Dopamine receptor antagonist* • *Serotonin receptor agonist^*	**Bipolar disorder** **Bradycardia** **Cardiac arrythmia** **Depression** **Headache** **Hypertension** **Migraine headache** **Schizophrenia**
*SLC6A4*	Solute carrier family 6 member 4	Agonist	**Vortioxetine** *Dopamine* *Dextromethorphan* *Tapentadol*	• Serotonin **receptor agonist** • *Dopamine receptor agonist* • *Glutamate receptor antagonist* • *Opioid receptor agonist* • *Sigma receptor agonist*	**Depression** *Acute pain* *Cough suppressant* *Headache* *Muscle pain* *Tremors* *Ventricular arrhythmias*
*PDE4C*	Phosphodiesterase 4 C	Agonist	**Ketotifen**	• **Phosphodiesterase inhibitor** • *Histamine receptor agonist* • *Leukotriene receptor antagonist*	**Itching**
*PDE4D*	Phosphodiesterase 4D	Antagonist	**Aminophylline** **Doxofylline** **Caffeine** **Dyphyllin** **Ketotifen** **Ibudilast** **Apremilast** **Dipyridamole** **Pentoxifylline** **Roflumilast** **Iloprost**	• **Phosphodiesterase inhibitor** *• Adenosine receptor antagonist* *• Histamine receptor agonist* *• Leukotriene receptor antagonist* *• Platelet aggregation inhibitor* *• Prostanoid receptor agonist*	**Asthma** **Bronchitis** **Chronic obstructive pulmonary disease** **Claudication** **Coronary artery disease (CAD)** **Drowsiness** **Emphysema** **Fatigue** **Hypertension** **Itching** **Peripheral artery disease (PAD)** **Psoriasis** **Psoriatic arthritis** **Pulmonary arterial hypertension (PAH)** **Stroke**
*PSMA5*	Proteasome subunit alpha 5	Antagonist	**Bortezomib** **Carfilzomib**	• **Proteasome inhibitor** *• NFkB pathway inhibitor*	**Multiple myeloma** **Mantle cell lymphoma (MCL)**
*THRB*	Thyroid hormone receptor beta	Agonist	**Levothyroxine** **Liothyronine** **Tiratricol**	• **Thyroid hormone receptor beta**	**Myxedema coma** **Hypothyroidism** **Refetoff syndrome**

Predicted nootropic function was obtained from gene expression association with general cognitive ability. MOA, drug names and drug indications were annotated via Broad Institute Connectivity MAP: Drug Re-Purposing hub. Labels in bold directly implicate the gene, while labels in italics indicate drugs and MOA that are consistent with the predicted nootropic function, but only indirectly implicate the gene.

*MOA* Mechanism of Action.

## Discussion

We report the largest joint GWAS analysis of GCA at an estimated sample size of 373,617. A total of 241 significant genetic loci were identified via MTAG procedures, of which 39 were not reported in the input cognitive GWASs, and 29 were fully novel to this report, despite substantial sample overlap between the two input GWAS. Notably, within these 29 loci are several genes that have been previously associated with psychiatric and brain related phenotypes, as well as underlying biological mechanisms that may underlie severe cognitive deficits (see Figs. S3–S30). Though not exhaustive, the list of such genes includes *CTNNA2, LMF1, ZNF536, BAIAP2*, and *GOSR1*, discussed below.

Catenin (Cadherin-Associated Protein), Alpha 2 (*CTNNA2*), encodes for brain expressed alpha-catenin crucial for synaptic contact, previously implicated with excitement seeking, risk-taking, hyperactivity, substance use, bipolar, and antisocial disorders [41]. *CTNNA2* was found to be neuronal-specific, and abundant in the dorsolateral prefrontal cortex and hippocampus in primate brains; *CTNNA2* and other catenin genes are involved in folding and lamination of the cerebral cortex [42]. Loss of function mutations within *CTNNA2* are thought to be one of the factors underlying pachygyria, characterized by loss of neurite stability and migration [43]. Lipase Maturation Factor 1 (*LMF1*) lipoproteins are mostly made in the brain; neurons and astrocytes coordinate lipoprotein metabolism within the brain [44] and evidence for lipoprotein lipase activity mediated by LMF genes implicate regulation of brain energy balance underlying cognition [45]. Zinc-Finger 536 (*ZNF536*) was identified as a candidate schizophrenia gene and a double-knockout zebrafish line shows behavioral and neuroanatomical (decreased forebrain volume) changes [46]. *ZNF536* has also been implicated in the maintenance of neural progenitor cells and neuronal differentiation within the prefrontal cortex [47, 48]. Brain-specific angiogenesis inhibitor 1-associated protein 2 (*BAIAP2*) is a multi-domain scaffolding and adapter protein implicated in the regulation of membrane and actin dynamics at subcellular structures, and is an abundant component of the postsynaptic density at excitatory synapses and an important regulator of actin-rich dendritic spines [49]. *BAIAP2* has been shown to be potentially involved in the etiology of attention deficit/hyperactivity disorder [50]. Golgi SNAP receptor complex member 1 (*GOSR1*) is involved in transport from the ER to the Golgi apparatus as well as in intra-Golgi transport [51]. The gene has been identified in large-scale genetics of brain phenotypes and is also reportedly associated with cognitive traits in follow-on analysis [52]. Similarly, evidence has also been reported that *GOSR1* is implicated in architecture of epigenetic and neuronal aging rates in human brain regions [53]. Further follow up is necessary to validate these genes in larger samples and biological experiments.

Gene property analysis revealed significant tissue expression overrepresented in CNS tissue compared with expression in other types of tissues, consistent with earlier reports [7–9, 39]. Notably, some of these genes appear significantly expressed during the prenatal state, indicating potential neurodevelopmental impact of genes associated with GCA. We focused on identifying genes associated with GCA that could be “actionable” in terms of identifying pharmacological agents that could be re-purposed for nootropic utilization based on GWAS results. In an earlier study, MAGMA pathway analysis was carried out on drug-based pathway annotations using a smaller GWAS of GCA [7], where we reported several T and L-type calcium channels as potential targets for nootropic agents. Here, we were able to leverage recent novel developments, including newly available brain eQTL data and complementary transcriptomic methodologies, enabling estimation of directionality (i.e., up- vs. down-regulation of expression) of gene effects on cognition. Notably, our study is the first cognitive GWAS to employ HEIDI, an approach that allows pleiotropy (either vertical or horizontal) to be differentiated from spurious associations due to linkage. HEIDI tests against the null hypothesis that a single causal variant affects both gene expression and trait variation, and so HEIDI-significant genes are less likely to be causal and require closer inspection and further biological experiments to unravel any true functional effects of the genes. Therefore, we have filtered gene results based on a nominal threshold of *P*_HEDI_ < 0.01. Additionally, several novel classes of gene sets, such as cell binding, cell metabolism, and cell structure not previously reported as associated with GCA, created an additional pool of genes available for further investigation. While many of the genes in these novel sets were not easily druggable, identification of methylation and chromatin binding gene sets further highlights the link between GCA, neurodevelopment, and psychopathology; loss of function variants in multiple genes in these sets result in increased risk for schizophrenia, autism, and other neurodevelopmental disorders [54–58].

The most crucial stage of the current report involved the identification of genes that are potential drug targets. Using filtering methods that were detailed earlier, the 76 “high-confidence” druggable genes were selected for further annotation. Of these, 16 genes were identified as “most likely druggable” based on their predicted function from eQTL results and the CMAP Drug Re-purposing database [36]. These selected genes could be further classified into broad gene classes (i) Serotonergic genes, (ii) Carbonic Anhydrase, (iii) Phosphodiesterase, (iv) Ion channel, (v) Glutamatergic/GABA-ergic, and (vi) Others (See Table 2); details of these gene classes are provided in the Extended Discussion in the supplement. Though we have focused the discussion of results explicitly on identifying potential targets for nootropic purposes, the converse could also be relevant—where there might be commonly administered drugs that appear to result in cognitive deficits. Indeed, we note that at least two drugs with known cognitive side effects, topiramate [59] and gabapentin [60], are predicted by our model to cause cognitive deficits.

The results here have generated leads for further investigation into potential drug targets and how they might subserve nootropic repurposing. However, there are limitations to the evidence that we report. First, although this report comprises the largest and most well-powered genome-wide analysis of GCA, there continues to be potential to expand sample size to increase power. The modest increase in novel loci reported in the current study could be accounted for by substantial sample overlap in the earlier GWAS reports. Nevertheless, it is notable that results of MTAG simulation of 75 and 88.9% sample overlap were largely similar to those reported in Turley et al., [30], which used height to show that MTAG was robust to 50% sample overlap. Here, under simulation conditions, we were able to show that MTAG can effectively control for inflation due to largely overlapping samples and is a useful tool for merging and combining summary statistics from large-scale GWAS into a single high-powered set of summary statistics. Relatedly, the present study focused on general cognitive ability, as this broad phenotype has the largest available sample size for GWAS; studies of individual cognitive subdomains (such as memory, processing speed, etc.) tend to be far less powered. Nevertheless, it is worth noting that there is some overlap of our results with prior, smaller studies of individual cognitive domains [61, 62]. As just one example, one of our short list of “most likely druggable” genes, *GRIN2A*, was previously reported to link schizophrenia with performance on an antisaccade task [63].

Additionally, the transcriptomic reference databases (e.g., PsychENCODE) that we employed are an order of magnitude larger than those previously available, but brain annotations remain somewhat smaller than other QTL annotations (e.g., blood). Second, identifying eQTL for a particular phenotype is challenging– as with most summary statistics approaches, it is not always possible to directly confirm that the proposed eQTL is necessarily leading to variation in the phenotype [64]. Direct experimentation is required to rule out potential extraneous factors that might be pleiotropic to both phenotypic variation and eQTL effects. Third, the issue of LD complexity within a GWAS region makes identification of a gene that is deemed associated with the phenotype challenging [65], further computational and functional work is needed to enhance the precision to which genes are prioritized or identified as truly functional within these genomic regions. Here, we attempted to identify functionally relevant genes by examining the convergence across a range of complementary methodologies to overcome some of the limitations noted above. In addition, we used the HEIDI test to explicitly exclude genes marked by linkage that might be inaccurately labeled as “causal”. Nevertheless, the challenge of regions of extensive LD encompassing many genes should be addressed in future studies, perhaps incorporating recently developed methods for examining three-dimensional properties of the genome [66]. Moreover, we attempted to combine results across multiple QTL tissues and annotations (Table S18), in order to match gene functions with drug MOA; however, we acknowledge that as yet there are no formal methodologies developed to statistically harmonize these signals.

We also observed several counter-intuitive findings with respect to directionality of effects: for example, with respect to carbonic anhydrase inhibition. It is plausible that many molecular functions in the brain observe either a U-shape or inverted U-shape curve, such that effects of up- or down-regulation are not strictly linear. Moreover, the results reported here are with reference to GWAS conducted in the general population and may be more complicated when applied to a disease population. For instance, calcium channel blockers might rescue cognitive impairments in schizophrenia, but blocking calcium channel function in the general population could be detrimental to synaptic function. At the same time, our GWAS cohorts included older adults, and some findings may be a function of cryptic pathologic processes occurring in these apparently normal subjects. Further work is necessary to replicate evidence reported here into disease populations, along with more precise data on biological mechanisms underlying cognitive function to ensure that compounds identified as nootropic are indeed applicable in disease contexts.

## Conclusions

We performed the largest genetic analysis for GCA. Aside from identifying 29 fully novel loci in the current study, the effort has included the most well powered analysis for identifying GCA-related genes that are “druggable” and for nominating potential drugs that could be repurposed for nootropic indications. Gene set analysis identified known neurodevelopmental and synaptic related pathways, as well as novel cell structure and binding pathways underlying GCA, which we exploited for drug identification purposes. Utilizing multiple chemoinformatic and drug repurposing databases, along with eQTL and GWAS data, we identified several gene classes contributing to GCA, including serotonergic and glutamatergic/GABA-ergic genes, voltage-gated ion channels, phosphodiesterase components, and carbonic anhydrases. Our efforts show that within these classes, specific drug candidates for nootropic repurposing appear most promising. Further work is necessary to confirm the role of these genes and receptors, to specify their biological mechanisms influencing cognition, and to consider potential CNS effects (including blood-brain barrier permeability) of the putative nootropic compounds nominated by this approach.

## Funding and Disclosures

This work has been supported by grants from the National Institutes of Health (R01 MH117646 to TL; R01 MH079800 and P50 MH080173 to AKM; R01 MH080912 to DCG; K23 MH077807 to KEB; K01 MH085812 to MCK). Data collection for the TOP cohort was supported by the Research Council of Norway, South-East Norway Health Authority, and KG Jebsen Foundation. The NCNG study was supported by Research Council of Norway Grants 154313/V50 and 177458/V50. The NCNG GWAS was financed by grants from the Bergen Research Foundation, the University of Bergen, the Research Council of Norway (FUGE, Psykisk Helse), Helse Vest RHF and Dr Einar Martens Fund. The Helsinki Birth Cohort Study has been supported by grants from the Academy of Finland, the Finnish Diabetes Research Society, Folkhälsan Research Foundation, Novo Nordisk Foundation, Finska Läkaresällskapet, Signe and Ane Gyllenberg Foundation, University of Helsinki, Ministry of Education, Ahokas Foundation, Emil Aaltonen Foundation. We thank the Lothian Birth Cohort participants and research team members. We thank the staff from the Wellcome Trust Clinical Research Facility at the Western General Hospital Edinburgh. Phenotype collection in the Lothian Birth Cohort 1921 was supported by the UK Biotechnology and Biological Sciences Research Council (BBSRC; 15/SAG09977), a Royal Society-Wolfson Research Merit Award (to Ian Deary), and The Chief Scientist Office of the Scottish Government (CZH/4/213, CZG/3/2/79, CZB/4/505, ETM/55). Phenotype collection in the Lothian Birth Cohort 1936 was supported by Age UK (The Disconnected Mind project). Genotyping of the cohorts was funded by the BBSRC (BB/F019394/1, 15/S18386). The work was undertaken by The University of Edinburgh Center for Cognitive Ageing and Cognitive Epidemiology (CCACE), part of the cross council Lifelong Health and Wellbeing Initiative (G0700704/84698, MR/K026992/1), for which funding from the BBSRC and Medical

Research Council (MRC) is gratefully acknowledged. WDH is supported by a grant from Age UK (Disconnected Mind Project). The CAMH work was supported by the CAMH Foundation and the Canadian Institutes of Health Research. The Duke Cognition Cohort (DCC) acknowledges K. Linney, J.M. McEvoy, P. Hunt, V. Dixon, T. Pennuto, K. Cornett, D. Swilling, L. Phillips, M. Silver, J. Covington, N. Walley, J. Dawson, H. Onabanjo, P. Nicoletti, A. Wagoner, J. Elmore, L. Bevan, J. Hunkin and R. Wilson for recruitment and testing of subjects. DCC also acknowledges the Ellison Medical Foundation New Scholar award AG-NS-0441-08 for partial funding of this study as well as the National Institute of Mental Health of the National Institutes of Health under award number K01MH098126. The UCLA Consortium for Neuropsychiatric Phenomics (CNP) study acknowledges the following sources of funding from the NIH: Grants UL1DE019580 and PL1MH083271 (RMB), RL1MH083269 (TDC), RL1DA024853 (EL) and PL1NS062410. The ASPIS study was supported by National Institute of Mental Health research grants R01MH085018 and R01MH092515 to Dr. Dimitrios Avramopoulos. Support for the Duke Neurogenetics Study was provided the National Institutes of Health (R01 DA033369 and R01 AG049789 to ARH) and by a National Science Foundation Graduate Research Fellowship to MAS. Recruitment, genotyping and analysis of the TCD healthy control samples were supported by Science Foundation Ireland (grants 12/IP/1670, 12/IP/1359 and 08/IN.1/B1916).

Data access for several cohorts used in this study was provided by the National Center for Biotechnology Information (NCBI) database of Genotypes and Phenotypes (dbGaP). dbGaP accession numbers for these cohorts were:

Cardiovascular Health Study (CHS): phs000287.v4.p1, phs000377.v5.p1, and phs000226.v3.p1

Framingham Heart Study (FHS): phs000007.v23.p8 and phs000342.v11.p8

Multi-Site Collaborative Study for Genotype-Phenotype Associations in Alzheimer’s Disease (GENADA): phs000219.v1.p1

Long Life Family Study (LLFS): phs000397.v1.p1

Genetics of Late Onset Alzheimer’s Disease Study (LOAD): phs000168.v1.p1

Minnesota Center for Twin and Family Research (MCTFR): phs000620.v1.p1

Philadelphia Neurodevelopmental Cohort (PNC): phs000607.v1.p1

The acknowledgment statements for these cohorts are found below:

**Framingham Heart Study**: The Framingham Heart Study is conducted and supported by the National Heart, Lung, and Blood Institute (NHLBI) in collaboration with Boston University (Contract No. N01-HC-25195 and HHSN268201500001I). This manuscript was not prepared in collaboration with investigators of the Framingham Heart Study and does not necessarily reflect the opinions or views of the Framingham Heart Study, Boston University, or NHLBI. Funding for SHARe Affymetrix genotyping was provided by NHLBI Contract N02-HL-64278. SHARe Illumina genotyping was provided under an agreement between Illumina and Boston University.

**Cardiovascular Health Study**: This research was supported by contracts HHSN268201200036C, HHSN268200800007C, N01-HC-85079, N01-HC-85080, N01-HC-85081, N01-HC-85082, N01-HC-85083, N01-HC-85084, N01-HC-85085, N01-HC-85086, N01-HC-35129, N01 HC-15103, N01 HC-55222, N01-HC-75150, N01-HC-45133, and N01-HC-85239; grant numbers U01 HL080295 and U01 HL130014 from the National Heart, Lung, and Blood Institute, and R01 AG-023629 from the National Institute on Aging, with additional contribution from the National Institute of Neurological Disorders and Stroke. A full list of principal CHS investigators and institutions can be found at https://chs-nhlbi.org/pi. This manuscript was not prepared in collaboration with CHS investigators and does not necessarily reflect the opinions or views of CHS, or the NHLBI. Support for the genotyping through the CARe Study was provided by NHLBI Contract N01-HC-65226. Support for the Cardiovascular Health Study Whole Genome Study was provided by NHLBI grant HL087652. Additional support for infrastructure was provided by HL105756 and additional genotyping among the African-American cohort was supported in part by HL085251, DNA handling and genotyping at Cedars-Sinai Medical Center was supported in part by National Center for Research Resources grant UL1RR033176, now at the National Center for Advancing Translational Technologies CTSI grant UL1TR000124; in addition to the National Institute of Diabetes and Digestive and Kidney Diseases grant DK063491 to the Southern California Diabetes Endocrinology Research Center.

**Multi-Site Collaborative Study for Genotype-Phenotype Associations in Alzheimer’s Disease**: The genotypic and associated phenotypic data used in the study were provided by the GlaxoSmithKline, R&D Limited. Details on data acquisition have been published previously in: Li H, Wetten S, Li L, St Jean PL, Upmanyu R, Surh L, Hosford D, Barnes MR, Briley JD, Borrie M, Coletta N, Delisle R, Dhalla D, Ehm MG, Feldman HH, Fornazzari L, Gauthier S, Goodgame N, Guzman D, Hammond S, Hollingworth P, Hsiung GY, Johnson J, Kelly DD, Keren R, Kertesz A, King KS, Lovestone S, Loy-English I, Matthews PM, Owen MJ, Plumpton M, Pryse-Phillips W, Prinjha RK, Richardson JC, Saunders A, Slater AJ, St George-Hyslop PH, Stinnett SW, Swartz JE, Taylor RL, Wherrett J, Williams J, Yarnall DP, Gibson RA, Irizarry MC, Middleton LT, Roses AD. Candidate single-nucleotide polymorphisms from a genome-wide association study of Alzheimer disease. Arch Neurol., Jan;65(1):45-53, 2008 (PMID: 17998437). Filippini N, Rao A, Wetten S, Gibson RA, Borrie M, Guzman D, Kertesz A, Loy-English I,

Williams J, Nichols T, Whitcher B, Matthews PM. Anatomically-distinct genetic associations of

APOE epsilon4 allele load with regional cortical atrophy in Alzheimer’s disease. Neuroimage,

Feb 1;44(3):724-8, 2009. (PMID: 19013250).

**Genetics of Late Onset Alzheimer’s Disease Study**: Funding support for the “Genetic Consortium for Late Onset Alzheimer’s Disease” was provided through the Division of Neuroscience, NIA. The Genetic Consortium for Late Onset Alzheimer’s Disease includes a genome-wide association study funded as part of the Division of Neuroscience, NIA. Assistance with phenotype harmonization and genotype cleaning, as well as with general study coordination, was provided by Genetic Consortium for Late Onset Alzheimer’s Disease. A list of contributing investigators is available at https://www.ncbi.nlm.nih.gov/projects/gap/cgi-bin/study.cgi?study_id=phs000168.v1.p1

**Long Life Family Study: Funding support for the Long Life Family Study** was provided by the Division of Geriatrics and Clinical Gerontology, National Institute on Aging. The Long Life Family Study includes GWAS analyses for factors that contribute to long and healthy life. Assistance with phenotype harmonization and genotype cleaning as well as with general study coordination, was provided by the Division of Geriatrics and Clinical Gerontology, National Institute on Aging. Support for the collection of datasets and samples were provided by Multicenter Cooperative Agreement support by the Division of Geriatrics and Clinical Gerontology, National Institute on Aging (UO1AG023746; UO1023755; UO1023749; UO1023744; UO1023712). Funding support for the genotyping which was performed at the Johns Hopkins University Center for Inherited Disease Research was provided by the National Institute on Aging, National Institutes of Health.

**Minnesota Center for Twin and Family Research**: This project was led by William G. Iacono, PhD. And Matthew K. McGue, PhD (Co-Principal Investigators) at the University of Minnesota, Minneapolis, MN, USA. Co-investigators from the same institution included: Irene J. Elkins, Margaret A. Keyes, Lisa N. Legrand, Stephen M. Malone, William S. Oetting, Michael B. Miller, and Saonli Basu. Funding support for this project was provided through NIDA (U01 DA 024417). Other support for sample ascertainment and data collection came from several grants: R37 DA 05147, R01 AA 09367, R01 AA 11886, R01 DA 13240, R01 MH 66140.

**Philadelphia Neurodevelopmental Cohort**: Support for the collection of the datasets was provided by grant RC2MH089983 awarded to Raquel Gur, MD, and RC2MH089924 awarded to Hakon Hakonarson, MD, PhD. All subjects were recruited through the Center for Applied Genomics at The Children’s Hospital in Philadelphia.

**Funding**: Primary support for this study came from the National Institute of Mental Health (R01 MH117646, PI: Lencz).

**Software and Online Resources:** Multi-Trait Analysis of GWAS (MTAG v1.08) https://github.com/omeed-maghzian/mtag, FDR Inverse Quantile Transformation (FIQT v1.0) https://github.com/bacanusa/FIQT, Functional mapping and annotation of genetic associations (FUMA v1.3.5) https://github.com/Kyoko-wtnb/FUMA-webapp, LD-hub v1.9.3 http://ldsc.broadinstitute.org/, Multi-marker Analysis of GenoMic Annotation (MAGMA v1.07b) https://ctg.cncr.nl/software/magma, S-PrediXcan/S-TissueXcan (v0.6) https://github.com/hakyimlab/MetaXcan; https://github.com/hakyimlab/MetaXcan/wiki, Summary-based Mendelian Randomization) and HEIDI (Heterogeneity in Dependent Instruments) tests (v1.02) https://cnsgenomics.com/software/smr/, LeafCutter (v1.0) https://github.com/davidaknowles/leafcutter, Drug-Gene Interaction database (DGIdb v.2) http://www.dgidb.org/, Psychoactive Drug Screening Database K_i_DB https://pdsp.unc.edu/databases/kidb.php, Broad Institute Connectivity Map, Drug Re-purposing Database https://www.broadinstitute.org/drug-repurposing-hub

Authors have no competing or conflicts of interests pertaining to the study.

## Supplementary information

Supplementary Material

STable2

STable3

STable4

STable5

STable6

STable7

STable8

STable9

STable10

STable11

STable12

STable13

STable14

STable15

STable16

STable17

STable19

Stable20

Stable21

STable18

## Data Availability

Cognitive GWASs utilized in the current manuscript are publicly available at https://ctg.cncr.nl/software/summary_statistics and https://www.ccace.ed.ac.uk/node/335. GWAS summary statistics that are reported in the current manuscript would be made available publicly upon publication of the manuscript. eQTL annotation data and drug annotation data are available in the respective weblinks included in the “Software and Online Resources” section.
