# Supplementary material for: Identifying nootropic drug targets via large-scale cognitive GWAS and transcriptomics
Source: Neuropsychopharmacology. 2021 May 25;46(10):1788–801. doi: 10.1038/s41386-021-01023-4 (PMC8357785; doi:10.1038/s41386-021-01023-4)
Supplement: Supplementary file 1 — Supplementary Material [file 41386_2021_1023_MOESM1_ESM.pdf]

Supplementary Materials

Supplementary Materials and Methods

Loci Discovery: MTAG-GWAS

We meta-analysed two sets of summary statistics using the Multi-Trait Analysis of GWAS (MTAG*[1]* v1.08). MTAG adjusts for sample overlap based on LD score regression, which is notable based on reported sample sizes, that approximately 89% of samples between Davies et al. *[2]* and Savage et al.*[3]* overlap. As part of the MTAG workflow, alleles in both sets of summary statistics were aligned against the 1000 genomes phase 3 version 5a reference panel*[4]*. We set filters for sample size N > 10,000, and variations with minor allele frequency > 0.001. To obtain a single output from MTAG, we set covariance of the two phenotypes to 1 and equivalent heritability across both phenotypes. This would approximate a fixed effect inverse variance meta-analysis but adjusting for sample overlaps across summary statistics inputs. We also carried out FDR Inverse Quantile Transformation analysis to account for potential Winner's Curse*[5]*. Following MTAG, significant loci were identified as follows: First, independent lead SNPs were identified using the clumping function in FUMA v1.3.5*[6]* with default parameters of $R^2 < 0.1$, 250*kb* window based on the 1000 Genomes Project Phase 3 European ancestry LD reference panel*[4]*. Independent loci were then identified by grouping SNPs that are within an LD of $R^2 > 0.6$ of the lead SNP. Finally, loci within 250kb of each other were merged into a single locus. GWAS significant threshold was set to $P < 5e\text{-}8$, which was also the threshold used by FUMA to identify the lead SNPs with *P*-values less than or equal to the GWAS significant threshold. SNPs in LD with the significant independent SNP were selected based on the secondary $P < 0.05$ threshold. The minor allele frequency threshold for SNPs to be included in annotation and prioritization was MAF > 0.01. We applied the default value of 10 kb for positional mapping of SNPs to genes, or functional consequences.

Winner's Curse Adjustment – FDR Inverse Quantile Transformation

To check for potential Winner's Curse in loci originally listed as significant in either Savage et al.,*[3]* and Davies et al.,*[2]* but no longer significant in the MTAG analysis, we first performed FDR Inverse Quantile Transformation (FIQT) on the Z scores of either of the discovery cohorts. Predicted Z-scores are calculated using the square root of sample size as a scaling factor as follows:

$$Z_{pred} = Z\mu * \sqrt{\frac{MTAG_N}{GWAS_N}}$$

Where, Z_{pred} is the predicted Z-scores without Winner's Curse, $Z\mu$ is the FIQT adjusted Z-scores, and $\sqrt{\frac{MTAG_N}{GWAS_N}}$ is the scaling factor assuming linear scaling based on sample size increase in fixed-effect meta-analysis as previously described*[5]*. To test if these results are potentially due to Winner's Curse, we performed FDR Inverse Quantile Transformation (FIQT) procedures on the input summary statistics.

MTAG simulation for sample overlaps at 75% and 88.9%

Due to large sample overlaps reported in the earlier cognitive GWAS*[2,3]*, it was necessary to examine how MTAG handles these overlaps in terms of potential inflation or deflation of summary statistics. We simulated genotypes for HapMap3 SNPs from 100,000 individuals using HAPGEN2*[7]* with 1000 Genomes Project European samples (N=503) as the reference panel. For each simulation replicate, we simulated an infinitesimal genetic architecture, assuming that the effect size of each SNP follows a standard normal distribution. The phenotype was generated by the sum of the genotypes of all SNPs, weighted by their simulated effect sizes, and adding a normally distributed noise term which fixed the SNP heritability at 0.15. We generated 20 simulates of the phenotype. For each simulation instance, we created subsamples from the 100,000 individuals using the first 80,000 (or 90,000) and last 80,000 (or 90,000) individuals of the data set, producing two scenarios with 75% (or 88.9%) sample overlap. First, we conducted GWAS on the main sample consisting of 100,000 individuals. Next, we conducted GWAS for each subsample to generate a pair of GWAS summary statistics. Lastly, we ran MTAG on the pair of GWAS summary statistics and compared MTAG z statistics against the z statistics from the GWAS conducted on the full sample.

Genome-wide characterization

To confirm that our genetic discoveries reflected brain-based biological traits underlying cognitive performance, a gene property analysis was used to screen gene-expression and localization in CNS tissues vs. all other biological tissue as implemented in MAGMA*[8]* utilizing GTEx v7 (http://www.gtexportal.org/home/datasets) tissues.

We carried out phenome-wide genetic correlation analysis using LD-hub*[9]* (v1.9.3) to determine and visualize the relationship between cognition and other psychiatric and physical traits.

Gene-based characterization

To identify potential functional genes that underpin genome-wide association results emanating from the MTAG analysis for GCA, several gene-based transcriptomics and pathway-based approaches were utilized to elucidate a set of putative genes that could be followed-up in subsequent analyses.

S-PrediXcan/S-TissueXcan Transcriptome-wide analysis of gene expression

To expand our analysis from SNPs/loci associations to the identification of putative causal genes, several methods were employed (See Workflow - Fig. 1 – Gene-Based Chracterization; Table S1). First, genetically regulated gene expression was imputed for MTAG meta-analysis using tissue models from GTExv7, which contains 48 different tissue types across 30 general tissue categories. The summary statistics from this meta-analysis were entered into the S-PrediXcan (Web app 18 Apr 2019) framework (https://github.com/hakyimlab/MetaXcan). S-PrediXcan computes gene-based associations where genetic effects on phenotypes are mediated through gene expression*[10]* (see also http://predictdb.org/). Next, we utilized S-TissueXcan to exploit the gene expression-mediated associations shared across multiple tissues

to enhance power for gene identification. We combined all S-PrediXcan results based on individual tissue types in GTEx v7 using S-TissueXcan. Gene-based p-value is computed via an omnibus test, which are then Bonferroni corrected. Both S-TissueXcan processing and post-processing pipelines are available online (https://github.com/hakyimlab/MetaXcan). We paid special attention to S-PrediXcan's brain tissue annotations in later stages of the analysis, extracting genes that are significantly associated with cognition after Bonferroni correction for each brain tissue within the GTEx database to lend post-hoc support for the gene-identification approaches.

Summary Statistics Based Mendelian Randomization (SMR and HEIDI)[11]

As a more conservative approach to transcriptomic-based gene identification, we utilized SMR (Summary-based Mendelian Randomization) and HEIDI (Heterogeneity in Dependent Instruments) tests*[11]* (v1.02). SMR uses a Mendelian Randomization (MR) approach where one or multiple SNPs could be used as instruments to identify gene expression effects on a given trait with estimated SNP-gene expression and SNP-phenotype effects. At the same time, the HEIDI test identifies SNP-gene expression effects and SNP-phenotype effects that are correlated with each other through LD rather than biologically related via pleiotropy or a causal pathway. We prioritized GWAS-identified genes for follow-up functional studies by including only genes with biologically related expression and phenotype effects (i.e., excluding genes with $P_{HEIDI} < 0.01$). For the SMR analyses, we utilized multiple transcriptomic reference datasets: (i) GTEx-brain eQTL data with estimated effective sample size (N) of 233, which includes an eQTL based meta-analysis of 10 brain regions from the GTEx, while correcting for sample overlap*[12,13]*; (ii) Brain-eMeta eQTL data with estimated effective N = 1,194, which includes an eQTL based meta-analysis of GTEx-brain, CommonMind Consortium, and xQTLServer (ROSMAP) datasets*[12]*; (iii) the PsychENCODE prefrontal cortex eQTL data (N = 1,866). Two sets of brain-based eQTL were generated from the PsychENCODE data based on earlier reports: (a) eQTL corrected for 50 Probabilistic Estimation of Expression Residuals (PEER), where only SNPs with expression association FDR <0.05 were included*[14]* and (b) eQTL corrected for 100 Hidden Covariates with Prior knowledge (HCP) included as covariates*[15]*. For brain-based eQTL datasets utilized by SMR and HEIDI, only SNPs within 1Mb of each probe were included as a proxy for *cis*-acting eQTL (See https://cnsgenomics.com/software/smr/#Overview). Both SMR and HEIDI p-values were Bonferroni corrected for multiple testing in 15,302 genes. Due to SMR's more conservative estimation of p-values, we also performed Benjamini-Hochberg false discovery rate (FDR) correction for SMR genes, after the primary analysis.

Brain-based eQTL mapping

eQTL mapping was carried out as part of the FUMA pipeline. Brain eQTL annotations were utilized for eQTL mapping. Databases used for eQTL mapping include: (i) BRAINEAC (http://www.braineac.org). A total of 134 neuropathologically confirmed control individuals of European descent from UK Brain Expression Consortium were included in the BRAINEAC data. All eQTLs with nominal p-value < 0.05 were identified in the cerebellar cortex, frontal cortex, hippocampus, inferior olivary nucleus, occipital cortex, putamen, substantia nigra, temporal cortex, thalamus, and white matter regions and based on averaged expression across all of them. (ii) GTEx v7: For eQTL mapping analysis, we chose brain tissue expression from

GTEX v7 and defined significant eQTLs as FDR (gene q-value) < 0.05. The gene FDR is pre-calculated by GTEx and every gene-tissue pair has a defined p-value threshold for eQTLs based on permutation. (iii) xQTLServer (http://mostafavilab.stat.ubc.ca/xqtl/): Expression of dorsolateral prefrontal cortex from 494 samples. (iv) Brain expression from 467 Caucasian samples available at the CommonMind Consortium (https://www.synapse.org//#!Synapse:syn5585484). Publicly available eQTLs from CMC are threshold by FDR into four groups: <0.2, <0.1, <0.05 and <0.01.

Finally, we mapped several novel forms of molecular quantitative trait loci (QTL). These novel QTLs include expression variation, splicing, and translation using post-mortem prefrontal cortex tissue data from the PsychENCODE/BrainGVEX project. In these samples, gene transcription and translation activities were assayed by RNA-sequencing (N=416) and ribosome profiling (N=192); Annotation data was available for novel QTLs with SNPs at P_{MTAG} < 1e-5: (i) gene expression variation QTL (evQTL) analysis tests for genetic loci that influence variance of expression level, using Bartlett's test*[16]* on the RNA-seq data; (ii) splicing QTL (sQTL) analysis captures the effects of genetic variations on RNA splicing, using leafcutter*[17]* on RNA-seq data (iii) ribosome occupancy QTL (rQTL) analysis identifies genetic variations that influence translation-related ribosome occupancy using Ribo-seq data; prior reports suggest that differences between transcription and translation QTLs may yield novel biological insights beyond standard eQTLs alone*[18,19]*. We focused on the cis-QTL by testing SNPs within 1 Mb of genes for the three molecular phenotypes. Significant QTL association was defined by FDR p value < 0.05. For this analysis, we carried out mean-variance QTL mapping, using on the double generalized linear model approach discussed in detail elsewhere*[16,20]*. In prior simulations, mean-variance approach to QTL mapping and associated permutation procedures have shown to be robust in reliably identifying QTL in face of variance heterogeneity. For comparison purposes, we also performed standard eQTL mapping on this dataset.

MAGMA Gene- and Gene Set-based association analysis

MAGMA*[8]* (v1.07) gene-based association tests were carried out as part of the FUMA pipeline. The MAGMA gene-based test combines individual SNP p-values in a pre-defined gene region into a gene-based p-value by calculating the mean chi-square statistics accounting for LD between SNPs and correcting for gene size. LD between SNPs within the genes is estimated based on the 1000 genomes phase 3 European ancestry panel. MAGMA competitive pathway analysis was conducted with results emerging from earlier MAGMA gene-based, S-PrediXcan/S-TissueXcan, and SMR/HEIDI analyses. Gene sets that were tested included custom-curated neurodevelopmental and other brain-related gene sets that had gone through stringent quality control in a study originally designed to interrogate rare variants in schizophrenia*[21]*. In the latter, pathways with more than 100 genes from Gene Ontology (release 146; June 22, 2015 release), KEGG (July 1, 2011 release), PANTHER (May 18, 2015 release), REACTOME (March 23, 2015 release), DECIPHER Developmental Disorder Genotype-Phenotype (DDG2P) database (April 13, 2015 release) and the Molecular Signatures Database (MSigDB) hallmark processes (version 4, March 26, 2015 release) were initially included. Additional gene sets were selected based on risk for schizophrenia and neurodevelopmental disorders, including those reported for schizophrenia rare variants*[22]* (translational targets of FMRP*[23,24]*, components of the post-synaptic density*[22,25]*, ion channel proteins*[22]*, components of the ARC, mGluR5, and NMDAR complexes*[22]*, proteins

at cortical inhibitory synapses*[26,27]*, targets of mir-137*[22]*, and genes near schizophrenia common risk loci*[22,28]*) and autism risk (These include: (1)targets of *CHD8*[29–31], (2) splice targets of RBFOX[31–33], (3) hippocampal gene expression networks*[34]*, (4) neuronal gene lists from the Gene2cognition database [http://www.genes2cognition.org]*[31]*, as well as (5) loss of function intolerant genes (pLI > 0.9 from the ExAC v0.3.1 pLI metric), (6) ASD exomes risk genes for FDR < 10% and 30%, and (7) ASD/developmental disorder *de novo* genes hit by a LoF or a LoF/missense *de novo* variant*[35,36]*). Brain-tissue expression gene-sets included the Brainspan RNA-seq dataset*[37]* and the GTEx v7 dataset*[13]*. We report significant gene sets that were associated with GCA to identify biological pathways putatively associated with GCA. Moreover, we use this information to further interrogate genes within these pathways with protein products that may serve as druggable targets, but which failed to attain genome-wide significance on their own. As such, we extracted nominally significant (p<.05) genes within the significant gene sets for further drug target annotations; this threshold was selected to strike a balance between potential false positive and false negative associations within gene sets that had already demonstrated association signal to GCA.

"Druggable" Gene Annotations

We identified a set of "druggable" gene targets derived from the Drug-Gene Interaction database (DGIdb v.2), Psychoactive Drug Screening Database K_iDB, and a recent review on "druggability"*[38]*. The "Druggable genome" as previously identified by Finan et al.,*[38]* includes 4,465 gene targets and is annotated into 3 Tiers based on "druggability" levels: (i) Tier 1 gene targets are those derived from FDA-approved compounds, or from compounds that are presently studied in clinical trials; (ii) Tier 2 gene targets include genes with high sequence similarity to Tier 1 proteins, or those that are targeted by small drug-like molecules; (iii) Tier 3 gene targets code for secreted and extracellular proteins, which also belong to "druggable" gene families. DGIdb v.2 integrates drug-gene interactions from 15 databases, including DrugBank and ChEMBL. The data is directly available as drug-gene pairs; the K_iDB provides K_i values for drug/target pairs and is particularly relevant for psychoactive drugs. Using filtering criterion previously reported by Gaspar and Breen*[39]* (K_iDB: "With non-empty K_i field", "Only Human", "K_i not superior or inferior to a value", "With molecule name", "With gene-name", "Unique pairs", "With range pK_i > 2"; DGIdb: "Number of unique gene-sets > 2"), we identified and updated 2,567 potential gene targets from the chemoinformatic databases. For further gene-target annotations, we took the intersection between genes extracted from the chemoinformatic database, and those reported in Finan et al*[38]*. This resulted in 1,876 genes for further annotations. At the final stage of the analysis we annotated high confidence genes using the Broad Institute Connectivity Map, Drug Re-purposing Database*[40]* which provides more in-depth details such as drug names, mechanism of action, and drug indications.

Extended Discussion: Review of Putative Drug Targets

Serotonergic Genes

The most novel and intriguing finding is identifying several serotonergic genes as relevant to cognitive function. These genes were not identified under genome-wide significant peaks, but rather emerged using our gene-set annotation strategy; therefore, some caution in interpretation should be exercised. Nevertheless, serotonergic mechanisms in cognition have support from several prior lines of research. For example, reduced serotonin may be linked to

cognitive disturbances and certain conditions such as Alzheimer's disease and mood disorder, and stimulating serotonin activity in depression may be beneficial to cognition independent of general relief of depressive symptoms[41–43]. However, antidepressants typically inhibit the serotonin transporter, while present results suggest that enhancing its function may have pro-cognitive effects. Perhaps more readily interpretable, results of the present study show generally that upregulation of HTR1D and downregulation of HTR5A are associated with enhanced cognitive function. One popular antidepressant, Vortioxetine, is a 5-HT1D agonist and demonstrates some evidence of pro-cognitive efficacy*[44]*. The triptans, a class of 5-HT1D agonists used for treatment of migraine, also demonstrated initial efficacy in rescuing migraine-induced cognitive deficits*[45]*. At the same time, antagonizing the 5-HT_{5A} receptor has showed cognitive enhancement in a ketamine-based rat model of cognitive dysfunction and negative symptoms of schizophrenia*[46]*. While ergot-derived migraine treatments with action at 5-HT5A have not shown evidence of cognitive benefit*[47]*, these agents tend to have complex actions at multiple serotonin (and other neurotransmitter) receptors.

Carbonic Anhydrase Genes

We are first to report evidence that carbonic anhydrase genes may be implicated in cognitive function. Carbonic anhydrase activity within the hippocampal neurons modulates GABA-ergic functions, altering sensitivity of the gating function for signal transfer through the hippocampal network*[48]*. Modifying the function of carbonic anhydrase in animal models improved learning abilities, and possible perception, processing and storing of temporally associated signals*[49]*. Some early reports have also speculated the role of zinc homeostasis being related to cognitive impairment in Alzheimer's disease[50–52]. While results of the present study suggest that inhibition of carbonic anhydrase activity may enhance cognition, pharmacologic evidence to date has supported the opposite conclusion. Specifically, carbonic anhydrase activation has shown enhancement in synaptic efficacy, spatial learning, memory, as well as object recognition in rodents*[49,53]*; in humans, topiramate, a carbonic anhydrase inhibitor has been associated with cognitive deterioration*[54]*. One mechanism by which carbonic anhydrase inhibition might improve cognitive function is in the context of amyloid pathology, which may be dependent on carbonic anhydrase activity.

Phosphodiesterase Genes

Phosphodieterases (PDEs) catalyze the only known reaction terminating cyclic nucleotide signals. As such, they are crucial regulators of physiological and pathophysiological mechanisms that underlie these processes. Here, we report that a class of PDE-4s*, PDE4D* and *PDE4C* demonstrate association with cognitive function. PDE4s are expressed in the cerebral cortex, hippocampus, hypothalamus, striatum, dopaminergic neurons within the substantia nigra, and astrocytes*[55,56]*. Inhibiting PDE4 increases the phosphorylation of CREB and hippocampal neurogenesis propagating antidepressant mimicking and memory-enhancing properties*[57,58]*.Previously reported evidence implicated PDE4s, in particular, *PDE4D,* in possessing pro-cognitive and neuro-protective properties after the infusion of Rolipram*[59,60]*. The development of therapeutic indications for Alzheimer's Disease, Huntington's disease, schizophrenia, depression and cognitive enhancement continues to be the subject of ongoing research*[61,62]*. Extensive discussion of *PDE4D,* previously discovered by large scale educational attainment GWAS, is reported elsewhere*[63]*. In the present study, improved eQTL mapping supports the nootropic function of inhibiting *PDE4D.* The role of *PDE4C* appears to be less understood. Earlier reports indicate that though *PDE4C* is expressed in the brain, limited to

the cortex, thalamic nuclei and cerebellum*[56]*. No evidence currently exists to show if activating *PDE4C* plays a conclusive role in rescuing cognitive deficits*[64]*.

Glutamatergic Genes

The role of excitatory glutamatergic and inhibitory GABA-ergic neurons are well researched in their relationship to cognitive function and presence in the brain*[65,66]*. Glutamate mediates fast synaptic transmission and plays a key role in long term potentiation*[67]*, synaptic plasticity, learning and memory, and other cognitive functions*[68]*. Extended glutamate stimulation can be damaging to neurons and give rise to excitotoxicity, regarded as a precursor mechanism to several neurodegenerative disorders*[69,70]*. Indirect modulation of the glutamatergic system via positive allosteric modulators of AMPAR have shown nootropic properties in laboratory animals and human patients[71–75]. Direct modulation of glutamatergic pathway via antagonists, co-agonizing the glycine site, potentiatiating the activity of agonists via polyamines, neurosteroids, and histamines for purpose of cognitive enhancement has also been explored*[76]*.

Here, we identified AMPA4 agonists as potential cognitive enhancement agents. Within the CMAP Drug Re-purposing database*[40]*, we identified Piracetam, a known nootropic as an acetylcholine agonist that appear to have shown evidence for improving cognitive function*[77]* via complex glutamatergic and calcium signaling pathways*[78]*. A counterintuitive result was that down regulation of eQTL for *GRIN2A* was related to higher cognitive function. However, it appears that there has been discussion of how low dose antagonism of glutamatergic receptors (N-methyl-D-aspartate: NMDA-R) might increase excitatory effects of glutamate neurons*[79]*. Supporting evidence for the precognitive effects of NMDAR antagonists like memantine has also been reported in animal models and humans*[80]*. Based on existing evidence, we also show that the *GRIN2A* gene might also be indirectly targeted by norepinephrine transporter inhibitors, serotonin-norepinephrine reuptake inhibitors, and calcium channel blockers (Table 2).

Voltage-gated Ion Channel Genes

Voltage-gated ion channels have originally been studied with respect to etiologies of excitability disorders of the heart and muscles. Nevertheless, there is currently emerging evidence for the role of calcium, sodium and potassium channels in the etiopathologies of neuropsychiatric disorders*[81]*. Ostensibly, these neuropsychiatric disorders and accompanying cognitive function deficits could be rescued by therapeutics aimed at targeting the underlying putative channelopathies*[82]*. Voltage-gated calcium channels increase periplasmic calcium concentrations, which triggers a downstream cascade of proteins involving ion channel function, vesicle docking and small molecule transport*[83]*. Calcium trafficking and signaling play a crucial role in cognitive function[84–91]. There is also evidence to suggest that voltage gated calcium channels are necessary for the function of dopaminergic neurons on mesolimbic and mesocortical regions*[92,93]*. Prior reports have suggested that blocking L-type calcium channels could be a viable strategy for Alzheimer's disease but noted the paradoxical effect that these channels also promotes synaptic plasticity and spatial memory*[94]*.

Here, we identify upregulation of *CACNA2D2* and *CACNG3* genes associated with cognitive function. Though calcium channel genes have been identified previously in both cognitive function and neuropsychiatric disease GWASs, work in identifying reliable compounds for calcium activation is relatively nascent. Existing drugs targeting calcium channel receptors are mainly antagonists There is evidence to suggest that indirect activation of calcium channel

genes via activating sarco-/ER Ca2+ ATPase 2 (SERCA) appear to be neuroprotective and enhance cognition and memory in Alzheimer's mouse model*[95]*. SERCA resides in the endoplasmic reticulum and its dysregulation is thought to affect cognitive function in Darier's disease, schizophrenia, Alzheimer's disease, and cerebral ischemia*[96]*. In the current report, adrenergic receptor agonists could also potentially play a role in activating calcium channel genes highlighted.

Current results also point to the potential role of the chloride voltage channel gene *CLCN2* as a potential gene target for cognitive enhancement. *CLCN2* plays a crucial role in background conductance, removing excess Cl- ions within pyramidal cells in the hippocampus, and regulates excitability in GABAergic interneurons*[97]*. Loss-of-function mutations in CLCN2 are associated with leukoencephalopathy*[98]*, and, controversially, with epilepsy*[99,100]*; therefore, it is plausible that activation of *CLCN2* might serve to enhance cognitive function. By contrast, gain of function mutations*[101]* are associated with primary aldosteronism and subsequent hypertension, without cognitive impairment. The only drug with such a function identified by CMAP search was lubiprostone*[102]*, which is utilized for constipation and has unknown activity in the CNS.

Several genes do not fall into clear categories but nonetheless are crucial in the context of cognitive function, I.e., *DPP4*, *THRB, PSMA5, DHODH2.* While little is known about potential cognitive functions of *DHODH* or *PSMA5*, we examine *DPP4* and *THRB* below.

Dipeptidyl Peptidase 4

Dipeptidyl peptidase IV (DPP-IV) is a serine protease is known to inactivate glucagon-like peptide-1 (GLP-1), pituitary adenylate cyclase-activating polypeptide (PACAP) and glucose-dependent insulinotropic peptide (GIP), which gives rise to pancreatic insulin secretion. Inhibition of DPP-IV enzyme activity via the gliptin class of medications has thus been widely utilized as a treatment option for diabetes*[103]*. However, aside from glucose control, animal studies have shown pro-neurogenic*[104]*, anti-inflammatory*[105]* and neuroplasticity*[106]* properties. DPP-4 inhibitors appear to improve glucose control and protect against worsening in cognitive functioning in older patients with type 2 diabetes*[106]*, and in some cases improve cognitive function*[107]*. Benefits of DPP-4 inhibition in the post-stroke recovery phase and long-term clinical outcome had also been extensively discussed*[108]*. Reports have also shown that linagliptin possess neuroprotective properties attributed to elevated levels of incretins in the brain*[109]*, while sitagliptin appear to regulate synaptic plasticity in AD mice via activating GLP-1 and BDNF-Trkb signaling*[110]*. Data from the current report suggest that downregulation of *DPP4* in is associated with better cognitive function, and therefore *DPP4* inhibitors have been identified as potential drug repurposing candidates for pro-cognitive investigation.

Thyroid Hormone Receptor Beta

Thyroid hormones (TH) has a vital function in neurodevelopment and its receptors known to regulate neurogenesis in the hippocampus, hypothalamus and subventricular zone[111–113]. In adults, hypothyroidism is related to depressive-like symptomatology, dementia, memory impairment, and psychomotor deficits*[114]*. These syndromes are thought to be mediated through serotonergic*[115]* and/or catecholaminergic*[116]* pathways. Treatment of hypothyroidism improved cognitive performance in a mouse model of Alzheimer's disease*[117]* and patients*[118]*. Evidence for thyroid hormones implicating learning and memory through

synaptic plasticity, neuronal cell differentiation and maturation had also been presented*[119]*. These evidences converge with the data presented in the current study showing that activating the thyroid hormone beta receptor would potentially yield nootropic effects.

Supplementary Figures

A.

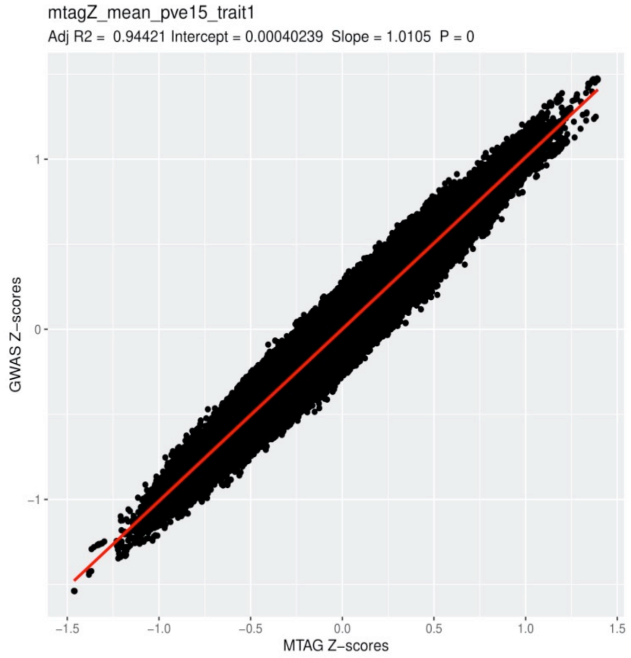

B.

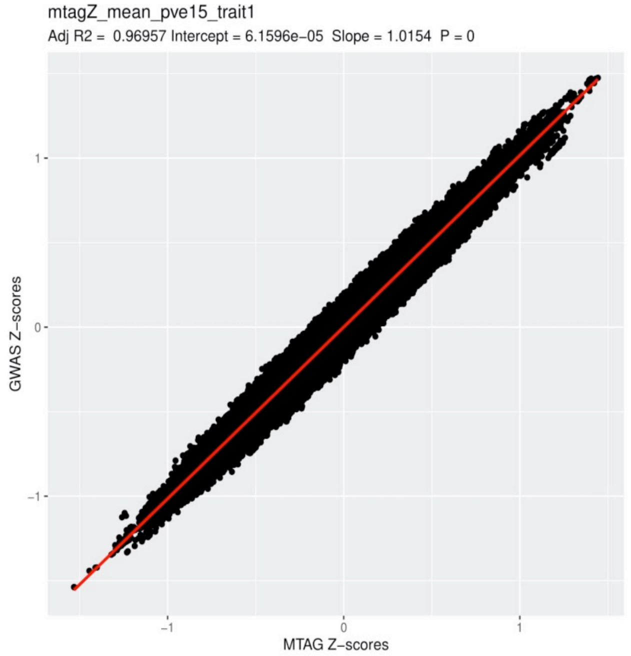

Fig S1. MTAG simulation results for 75% and 88.9% sample overlap.

A. MTAG simulation for 75% sample overlap; B. MTAG simulation for 80% sample overlap.

Note: GWAS Z-scores: GWAS association Z scores from main sample; MTAG Z-scores are from MTAG results of subsamples in A, and B.

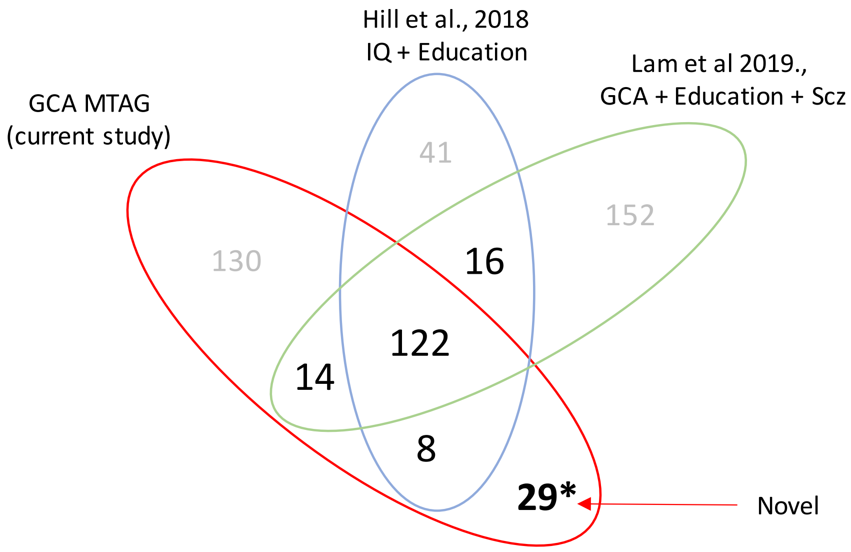

Fig S2. Overlapping Loci between MTAG GCA (Current Study), Hill et al., 2018 and Lam et al., 2019.

Note: Hill et al., 2018 "A combined analysis of genetically correlated traits identifies 187 loci and a role for neurogenesis and myelination in intelligence"; Pleiotropic Meta-Analysis of Cognition, Education, and Schizophrenia Differentiates Roles of Early Neurodevelopmental and Adult Synaptic Pathways

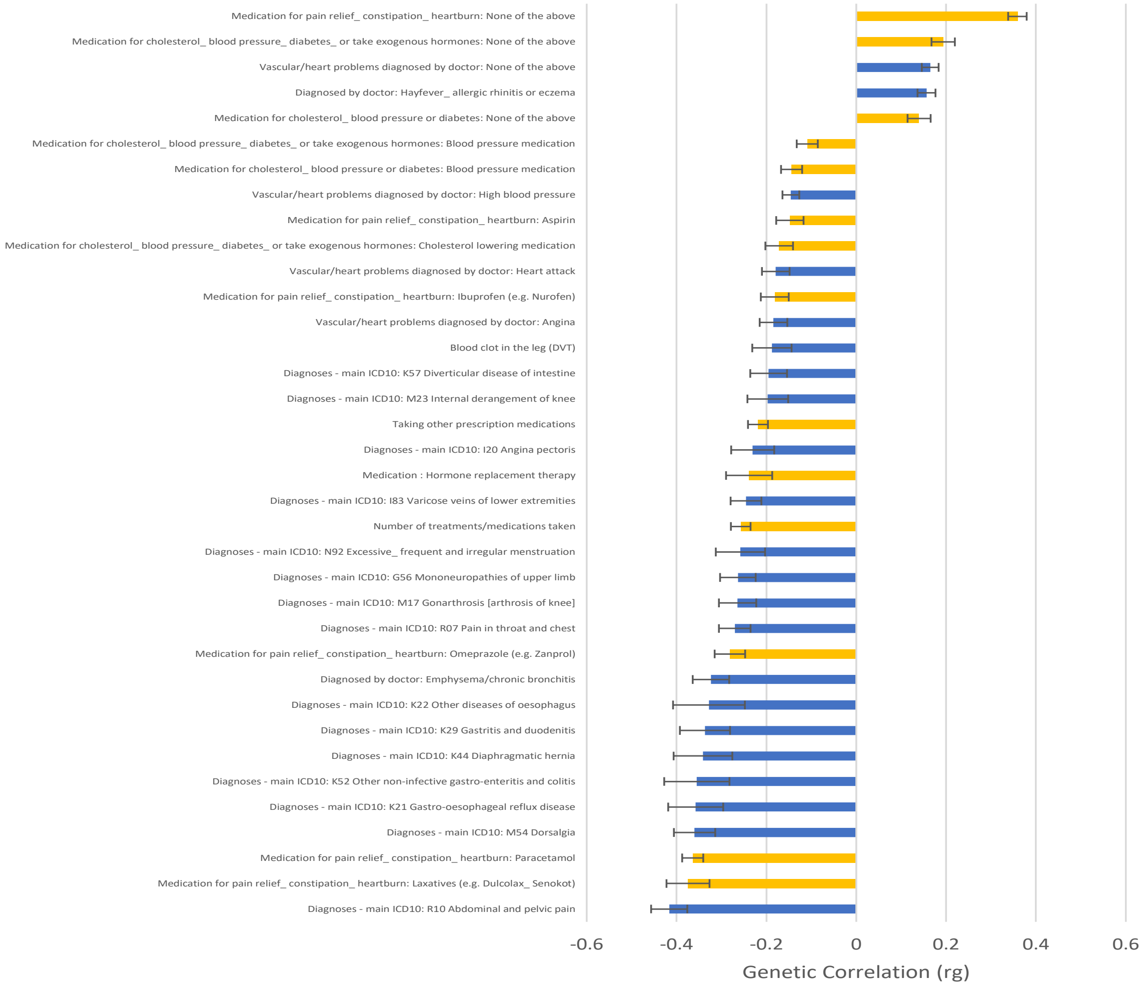

Fig S3. Genetic Correlations for UK Biobank ICD-10 and Medication phenotypes with GCA

Error bars denote standard errors. Yellow bars denote medication phenotypes. Blue bars denote ICD-10 phenotypes.

Fig S4-31. 29 Independent regions not previously associated with cognition

State No.	Mnemonic	Description
1	TssA	Active TSS
2	TssAFlnk	Flanking Active TSS
3	TxFlnk	Transcr. at gene 5' and 3'
4	Tx	Strong transcription
5	TxWk	Weak transcription
6	EnhG	Genic enhancers
7	Enh	Enhancers
8	ZNF/Rpts	ZNF genes & repeats
9	Het	Heterochromatin
10	TssBiv	Bivalent/Poised TSS
11	BivFlnk	Flanking Bivalent TSS/Enh
12	EnhBiv	Bivalent Enhancer
13	ReprPC	Repressed PolyComb
14	ReprPCWk	Weak Repressed PolyComb
15	Quies	Quiescent/Low

A ChromHMM model applicable to all 127 epigenomes was learned by virtually concatenating consolidated data corresponding to the core set of 5 chromatin marks assayed in all epigenomes (H3K4me3, H3K4me1, H3K36me3, H3K27me3, H3K9me3). The model was trained on 60 epigenomes with highest-quality data, which provided sufficient coverage of the different lineages and tissue types. The ChromHMM parameters used were as follows: Reads were shifted in the 5 to 3 direction by 100 bp. For each consolidated ChIP-seq dataset, read counts were computed in non-overlapping 200 bp bins across the entire genome. Each bin was discretized into two levels, 1 indicating enrichment and 0 indicating no enrichment. The binarization was performed by comparing ChIP-seq read counts to corresponding whole-cell extract control read counts within each bin and using a Poisson p-value threshold of 1e-4 (the default discretization threshold in ChromHMM). We trained several models in parallel mode with the number of states ranging from 10 states to 25 states. We decided to use a 15-state model for all further analyses since it captured all the key interactions between the chromatin marks, and because larger numbers of states did not capture sufficiently distinct interactions. The trained model was then

used to compute the posterior probability of each state for each genomic bin in each reference epigenome. The regions were labeled using the state with the maximum posterior probability.

EID	Color	Standardized Epigenome Name
E054		Ganglion Eminence derived primary cultured neurospheres
E053		Cortex derived primary cultured neurospheres
E071		Brain Hippocampus middle
E074		Brain Substantia Nigra
E068		Brain Anterior Caudate
E069		Brain Cingulate Gyrus
E072		Brain Inferior Temporal Lobe
E067		Brain Angular Gyrus
E073		Brain Dorsolateral Prefrontal Cortex
E070		Brain Germinal Matrix
E082		Fetal brain Female
E081		Fetal brain Male
E125		NH-A Astrocytes Primary Cells

Supplementary Tables

Table S1. Methodological Overview and Results Roadmap

Results	Methodology Outline	Software/Algorithm/Pipeline
Fig. 2A, B, C, D<br>Fig S4-31	Loci Discovery: MTAG-GWAS	Multi-Trait Analysis of GWAS (MTAG).
Table S2<br><br>Table S3	Clumping approach for independent significant SNPs. Merge independent loci identified by FUMA based on physical distance threshold of 250kb.	Functional Mapping and Annotation (FUMA)
Table S4	Winner's Curse Adjustment – FDR Inverse Quantile Transformation	FIQT
Fig S1A, B	MTAG simulation for sample overlaps at 75% and 88.9%	HAPGEN2<br>Plink 1.90<br>MTAG
	Genome-wide characterization	
Fig 2E, F	MAGMA gene property analysis – eQTL screen	FUMA - MAGMA gene property analysis
Table S5<br>Fig. S3	Genetic Correlation	LDSC/LD-hub
	Gene-Based Characterization of GCA MTAG genome-wide results	
Table S6	Joint Transcriptomic modeling of overall gene expression in GTEXv7 tissues	S-TissueXcan
Table S7a to S7k	Transcriptome wide analysis for gene expression in brain tissues	S-Predixcan
Table S8<br>Table S9	Summary Statistics Mendelian Randomization to identify putative causal genes implicating genes expression and GCA/Heterogeneity in dependent instrument test to de-prioritize genes implicated in high LD regions	SMR/HEIDI v1.02
	Brain-based molecular QTL mapping	
Table S10	eQTL mapping based on brain tissue expression within GTEX, BrainEAC, CMC, xQTLServer.	FUMA
Table S11<br>Table S12<br>Table S13<br>Table S14a to S14c	Molecular mapping of postmortem pre-frontal cortex tissue data of PsychENCODE/ BrainGVEX project: Four molecular phenotypes were used to further annotate results of GCA (i) expression variant QTL (ii) ribosome occupancy QTL (iii) expression QTL (iv) splicing QTL	Leafcutter<br>vQTL<br>Colocalization

Table S1 (cont'd). Methodological Overview and Analytic Approaches

Analysis	Methodology	Software/Algorithm/Pipeline
Table S15<br><br>Table 1a,b,c<br>Table S16	Gene-based and pathway identification<br><br>Gene Mapping of MTAG results<br><br>MAGMA Gene Set Analysis<br>- Nominally significant genes from multiple testing adjusted significant pathways	MAGMA<br><br>Gene Set Annotations<br>(See Materials and Methods – MAGMA Gene – and Gene Set – based association analysis)
Table S17<br><br>Table S18<br><br>Table S19<br>Table 2<br>Fig. 5	'Druggable' Gene Annotations<br><br>Drug gene-target annotations are curated from chemoinformatic databases. These genes have been previously identified to be functional and can be quickly repurposed for pharmacological investigation.<br><br>Combining gene results from Gene-Based Characterization with Drug Repurposing annotations<br><br>Identifying "High Confidence" druggable genes	Drug gene-targets curated from Finan et al., 2017, DGIdb v.2, K_iDB<br>1,876 genes that were 'druggable' after filtering<br><br>CMAP-DR

Table S2. Candidate SNPs within independent loci

Note: Savage.P : GWAS P-values reported by Savage et al; Savage.Z: GWAS Z-scores from Savage et al; Savage.N: Sample sizes reported by Savage et al; Davies.P : GWAS P-values reported by Davies et al; Davies.Z: GWAS Z-scores from Davies et al; Davies.N: Sample sizes derived from Davies et al; Savage.+/-: effect direction from Z-scores from Savage et al; Davies.+/-: effect direction from Z-scores from Davies et al; mtag.n.lower: lower bound estimated sample sizes by MTAG; mtag.n.upper bound estimated sample sizes by MTAG; mtag.frq: allele frequency based on MTAG; mtag_z: Z-score output from MTAG; mtag_pval: MTAG p-values.

Table S3. Genomic Loci and Top SNP within loci for MTAG analysis

Note: Savage.P : GWAS P-values reported by Savage et al; Savage.Z: GWAS Z-scores from Savage et al; Davies.P : GWAS P-values reported by Davies et al; Davies.Z: GWAS Z-scores from Davies et al; MTAG.SNP: SNP RSID output from MTAG; MTAG.A1/MTAG.A2: Harmonized allele outputs from MTAG; MTAG.Z: Z-score output from MTAG; MTAG.P: P-value output from MTAG.

Table S4. Winner's Curse Adjustment via FDR Inverse Quantile Transformation

Note: Z_{FIQT} : FIQT adjusted scores. These are Z-scores downweighted by the FIQT algorithm. We indicate Z scores from GWASs from either Davies or Savage et al; $Z_{predicted\text{-}Davies^\wedge}$: These are

the adjusted Z scores, assuming no winner's curse in Davies; $Z_{predicted\text{-}Savage^{\wedge}}$: These are the adjusted Z scores, assuming no winner's curse in Savage.

Table S5. Genetic correlations carried out via LD-hub v1.9.3

Table S6. S-TissueXcan Transcriptomic Wide Identified Genes

Note: p.adj: Bonferroni adjusted p-values for S-TissueXcan; p_i_best: smallest p-value for tissue type; t_i_best: tissue that has the smallest p-value; p_i_worst: largest p-value for tissue type; t_i_worst: tissue that has the largest p-value

Table S7. S-PrediXcan results for brain tissue

Table S7a : S-PrediXcan results : Nucleas Accumbens

Table S7b : S-PrediXcan results : Amygdala

Table S7c : S-PrediXcan results : Anterior Cingulate Cortex

Table S7d : S-PrediXcan results : Cerebellum

Table S7e : S-PrediXcan results : Cerebellar Hemisphere

Table S7f : S-PrediXcan results : Cortex

Table S7g : S-PrediXcan results : Frontal Cortex

Table S7h : S-PrediXcan results : Hippocampus

Table S7i : S-PrediXcan results : Hypothalamus

Table S7j : S-PrediXcan results : Putamen

Table S7k : S-PrediXcan results : Consolidated Bonferroni P-value Corrected S-Predixcan Gene List

Note: P.adj: Bonferroni adjusted p-values

Table S8. SMR and HEIDI analysis for SNP-Gene Mendelian Randomization

Note: p_SMR: SMR p-values; p_SMR_multi: multi-variant SMR p-values; p_HEIDI: HEIDI p-values; *_adj: bonferroni adjusted p-values; Bemeta: Brain-eMeta annotations; PsychEncode PEER: PsychENCODE data corrected for Probabilistic Estimation of Expression Residuals; PsychEncode HCP: PsychENCODE data corrected for Hidden Covariates with Prior Knowledge; Gtex: 10 brain regions from GTEx

Table S9. SMR and HEIDI results FDR corrected Gene Lists

Note: p_msmr: multi-variant SMR p-values; p_heidi: HEIDI p-values; B& H q-value: Benjamini & Hochberg q

Table S10. FUMA eQTL Mapping of Brain Expressed Genes

Table S11. PsychEncode/BrainGVEX Gene Expression QTL

Table S12. PsychEncode/BrainGVEX Ribosomal Occupancy QTL

Table S13. PsychEncode/BrainGVEX Expression Variation QTL

Table S14. PsychEncode/BrainGVEX Splicing QTL

Table S14a. PsychEncode/BrainGVEX Splicing QTL Leafcutter Results

Table S14b. VEP annotations for Splicing Variants

Table S14c. Splice Variant Gene List for Protein Coding Genes represented in MTAG results

Note: FDR: FDR p-values; qtl_p: qtl p-values; mtag_freq: allele frequencies based on MTAG output; mtag_beta: MTAG effect size; mtag_se: standard error of MTAG effect size; mtag_pval: MTAG p-values

Table S15. MAGMA gene based association test results
***Note:* P.Adj: Bonferroni adjusted p-values**

Table S16. MAGMA pathway analysis with gene results and druggability tier annotations
***Note:* Gene SET p-values have been Bonferroni adjusted**

Table S17. Drug Annotations from DGIdb, KI and Finan et al., 2017

Table S18. Candidate 'Druggable' Genes with gene expression profiles, and drug-disease indications from CMAP/CLUE

Note: Gene identification approaches: These include various strategies to identify genes that are associated with cognitive ability; Drug Targets: These are obtained from filtered drug targets; S-TissueXcan.GTEX7: significant genes identified by S-TissueXcan; Spredixcan.Brain (post-hoc):

Nominally associated genes within S-PrediXcan brain tissues; SMR.Brain.Bonferroni: SMR significant genes after Bonferroni correction; SMR.Brain.FDR (post-hoc): SMR significant genes after FDR correction; HEIDI.(p < 0.01): HEIDI genes at P < 0.01 threshold across annotations; MAGMA.Gene.Chisq: Bonferroni corrected significant MAGMA genes from gene association test; MAGMA.pathways: nominally significant genes within Bonferroni corrected significant MAGMA pathways; FUMA.eQTL.Brain: Significant genes from eQTL mapping via the FUMA pipeline; rQTL.PsychEnc.mvQTL: significant PsychENCODE ribosomal occupancy qtl genes; eQTL.PsychEnc.mvQTL: significant PsychENCODE eqtl genes; evQTL.PsychEnc.mvQTL: significant PsychENCODE expression variation qtl genes; sQTL.PsychEnc.mvQT: significant PsychENCODE splicing qtl genes; QTL directions: these effect directions based on eQTL results; CMAP/CLUE filters: annotations from CMAP-DR database, these include counts of drugs within development phase, (e.g. Launch, Phase1, Phase 2, …), and total drugs for specified indications (e.g. neurology/psychiatry, dermatology, cardiology, …).

Table S19. CMAP Drug Repurposing Database Annotations for 'High Confidence' Genes

Note: MOA: Mechanism of Action; Drug For: Drug indications; Predicted Function: from eQTL effect directions with general cognitive ability.

Table S20. Cell Specific Follow-up lookups of Top 76 Candidate Target Genes
Note: Genes highlighted in red are prioritized for nootropic repurposing

Table S21. Follow-up lookups of Developmental Epochs of Top 76 Candidate Target Genes
Note: Genes highlighted in red are prioritized for nootropic repurposing

References

1. Turley P, Walters RK, Maghzian O, Okbay A, Lee JJ, Fontana MA, et al. Multi-trait analysis of genome-wide association summary statistics using MTAG. Nat Genet. 2018;50:229–237.
2. Davies G, Lam M, Harris SE, Trampush JW, Luciano M, Hill WD, et al. Study of 300,486 individuals identifies 148 independent genetic loci influencing general cognitive function. Nature Communications. 2018;9:2098.
3. Savage JE, Jansen PR, Stringer S, Watanabe K, Bryois J, Leeuw CA de, et al. Genome-wide association meta-analysis in 269,867 individuals identifies new genetic and functional links to intelligence. Nature Genetics. 2018;50:912–919.
4. The 1000 Genomes Project Consortium. A global reference for human genetic variation. Nature. 2015;526:68–74.
5. Bigdeli TB, Lee D, Webb BT, Riley BP, Vladimirov VI, Fanous AH, et al. A simple yet accurate correction for winner's curse can predict signals discovered in much larger genome scans. Bioinformatics. 2016;32:2598–2603.
6. Watanabe K, Taskesen E, Bochoven A, Posthuma D. Functional mapping and annotation of genetic associations with FUMA. Nature Communications. 2017;8:1826.
7. Su Z, Marchini J, Donnelly P. HAPGEN2: simulation of multiple disease SNPs. Bioinformatics. 2011;27:2304–2305.
8. de Leeuw CA, Mooij JM, Heskes T, Posthuma D. MAGMA: Generalized Gene-Set Analysis of GWAS Data. PLoS Comput Biol. 2015;11.
9. Zheng J, Erzurumluoglu AM, Elsworth BL, Kemp JP, Howe L, Haycock PC, et al. LD Hub: a centralized database and web interface to perform LD score regression that maximizes the potential of summary level GWAS data for SNP heritability and genetic correlation analysis. Bioinformatics. 2017;33:272–279.
10. Barbeira AN, Dickinson SP, Bonazzola R, Zheng J, Wheeler HE, Torres JM, et al. Exploring the phenotypic consequences of tissue specific gene expression variation inferred from GWAS summary statistics. Nat Commun. 2018;9:1825.
11. Zhu Z, Zhang F, Hu H, Bakshi A, Robinson MR, Powell JE, et al. Integration of summary data from GWAS and eQTL studies predicts complex trait gene targets. Nat Genet. 2016;48:481–487.
12. Qi T, Wu Y, Zeng J, Zhang F, Xue A, Jiang L, et al. Identifying gene targets for brain-related traits using transcriptomic and methylomic data from blood. Nat Commun. 2018;9:2282.
13. GTEx Consortium, Laboratory, Data Analysis &Coordinating Center (LDACC)—Analysis Working Group, Statistical Methods groups—Analysis Working Group, Enhancing GTEx (eGTEx) groups, NIH Common Fund, NIH/NCI, et al. Genetic effects on gene expression across human tissues. Nature. 2017;550:204–213.
14. Wang D, Liu S, Warrell J, Won H, Shi X, Navarro FCP, et al. Comprehensive functional genomic resource and integrative model for the human brain. Science. 2018;362.
15. Gandal MJ, Zhang P, Hadjimichael E, Walker RL, Chen C, Liu S, et al. Transcriptome-wide isoform-level dysregulation in ASD, schizophrenia, and bipolar disorder. Science. 2018;362.
16. Corty RW, Valdar W. vqtl: An R Package for Mean-Variance QTL Mapping. G3 (Bethesda). 2018;8:3757–3766.
17. Li YI, Knowles DA, Humphrey J, Barbeira AN, Dickinson SP, Im HK, et al. Annotation-free quantification of RNA splicing using LeafCutter. Nat Genet. 2018;50:151–158.
18. Yao C, Chen G, Song C, Keefe J, Mendelson M, Huan T, et al. Genome-wide mapping of plasma protein QTLs identifies putatively causal genes and pathways for cardiovascular disease. Nat Commun. 2018;9:3268.
19. Chick JM, Munger SC, Simecek P, Huttlin EL, Choi K, Gatti DM, et al. Defining the consequences of genetic variation on a proteome-wide scale. Nature. 2016;534:500–505.

20. Corty RW, Kumar V, Tarantino LM, Takahashi JS, Valdar W. Mean-Variance QTL Mapping Identifies Novel QTL for Circadian Activity and Exploratory Behavior in Mice. G3 (Bethesda). 2018;8:3783–3790.
21. Singh T, Walters JTR, Johnstone M, Curtis D, Suvisaari J, Torniainen M, et al. The contribution of rare variants to risk of schizophrenia in individuals with and without intellectual disability. Nat Genet. 2017;49:1167–1173.
22. Purcell SM, Moran JL, Fromer M, Ruderfer D, Solovieff N, Roussos P, et al. A polygenic burden of rare disruptive mutations in schizophrenia. Nature. 2014;506:185–190.
23. Darnell JC, Van Driesche SJ, Zhang C, Hung KYS, Mele A, Fraser CE, et al. FMRP stalls ribosomal translocation on mRNAs linked to synaptic function and autism. Cell. 2011;146:247–261.
24. Ascano M, Mukherjee N, Bandaru P, Miller JB, Nusbaum JD, Corcoran DL, et al. FMRP targets distinct mRNA sequence elements to regulate protein expression. Nature. 2012;492:382–386.
25. Kirov G, Pocklington AJ, Holmans P, Ivanov D, Ikeda M, Ruderfer D, et al. De novo CNV analysis implicates specific abnormalities of postsynaptic signalling complexes in the pathogenesis of schizophrenia. Mol Psychiatry. 2012;17:142–153.
26. Heller EA, Zhang W, Selimi F, Earnheart JC, Ślimak MA, Santos-Torres J, et al. The Biochemical Anatomy of Cortical Inhibitory Synapses. PLOS ONE. 2012;7:e39572.
27. Pocklington AJ, Rees E, Walters JTR, Han J, Kavanagh DH, Chambert KD, et al. Novel Findings from CNVs Implicate Inhibitory and Excitatory Signaling Complexes in Schizophrenia. Neuron. 2015;86:1203–1214.
28. PGC2-SCZ. Biological insights from 108 schizophrenia-associated genetic loci. Nature. 2014;511:421–427.
29. Sugathan A, Biagioli M, Golzio C, Erdin S, Blumenthal I, Manavalan P, et al. CHD8 regulates neurodevelopmental pathways associated with autism spectrum disorder in neural progenitors. Proc Natl Acad Sci USA. 2014;111:E4468-4477.
30. Cotney J, Muhle RA, Sanders SJ, Liu L, Willsey AJ, Niu W, et al. The autism-associated chromatin modifier CHD8 regulates other autism risk genes during human neurodevelopment. Nature Communications. 2015;6:6404.
31. De Rubeis S, He X, Goldberg AP, Poultney CS, Samocha K, Cicek AE, et al. Synaptic, transcriptional and chromatin genes disrupted in autism. Nature. 2014;515:209–215.
32. Weyn-Vanhentenryck SM, Mele A, Yan Q, Sun S, Farny N, Zhang Z, et al. HITS-CLIP and integrative modeling define the Rbfox splicing-regulatory network linked to brain development and autism. Cell Rep. 2014;6:1139–1152.
33. Fogel BL, Wexler E, Wahnich A, Friedrich T, Vijayendran C, Gao F, et al. RBFOX1 regulates both splicing and transcriptional networks in human neuronal development. Hum Mol Genet. 2012;21:4171–4186.
34. Johnson MR, Shkura K, Langley SR, Delahaye-Duriez A, Srivastava P, Hill WD, et al. Systems genetics identifies a convergent gene network for cognition and neurodevelopmental disease. Nat Neurosci. 2016;19:223–232.
35. Sanders SJ, He X, Willsey AJ, Ercan-Sencicek AG, Samocha KE, Cicek AE, et al. Insights into Autism Spectrum Disorder Genomic Architecture and Biology from 71 Risk Loci. Neuron. 2015;87:1215–1233.
36. Deciphering Developmental Disorders Study. Large-scale discovery of novel genetic causes of developmental disorders. Nature. 2015;519:223–228.
37. Kang HJ, Kawasawa YI, Cheng F, Zhu Y, Xu X, Li M, et al. Spatio-temporal transcriptome of the human brain. Nature. 2011;478:483–489.
38. Finan C, Gaulton A, Kruger FA, Lumbers RT, Shah T, Engmann J, et al. The druggable genome and support for target identification and validation in drug development. Sci Transl Med. 2017;9.
39. Gaspar HA, Breen G. Drug enrichment and discovery from schizophrenia genome-wide association results: an analysis and visualisation approach. Scientific Reports. 2017;7:12460.

40. Corsello SM, Bittker JA, Liu Z, Gould J, McCarren P, Hirschman JE, et al. The Drug Repurposing Hub: a next-generation drug library and information resource. Nat Med. 2017;23:405–408.
41. Prado CE, Watt S, Crowe SF. A meta-analysis of the effects of antidepressants on cognitive functioning in depressed and non-depressed samples. Neuropsychol Rev. 2018;28:32–72.
42. Al-Sukhni M, Maruschak NA, McIntyre RS. Vortioxetine : a review of efficacy, safety and tolerability with a focus on cognitive symptoms in major depressive disorder. Expert Opin Drug Saf. 2015;14:1291–1304.
43. Schmitt J a. J, Wingen M, Ramaekers JG, Evers E a. T, Riedel WJ. Serotonin and human cognitive performance. Curr Pharm Des. 2006;12:2473–2486.
44. Rosenblat JD, Kakar R, McIntyre RS. The Cognitive Effects of Antidepressants in Major Depressive Disorder: A Systematic Review and Meta-Analysis of Randomized Clinical Trials. Int J Neuropsychopharmacol. 2015;19.
45. Edwards KR, Rosenthal BL, Farmer KU, Cady RK, Browning R. Evaluation of sumatriptan-naproxen in the treatment of acute migraine: a placebo-controlled, double-blind, cross-over study assessing cognitive function. Headache. 2013;53:656–664.
46. Nikiforuk A, Hołuj M, Kos T, Popik P. The effects of a 5-HT5A receptor antagonist in a ketamine-based rat model of cognitive dysfunction and the negative symptoms of schizophrenia. Neuropharmacology. 2016;105:351–360.
47. Evers S, Rüschenschmidt J, Frese A, Rahmann A, Husstedt I-W. Impact of antimigraine compounds on cognitive processing: a placebo-controlled crossover study. Headache. 2003;43:1102–1108.
48. Sun M-K, Alkon DL. Carbonic anhydrase gating of attention: memory therapy and enhancement. Trends Pharmacol Sci. 2002;23:83–89.
49. Sun MK, Alkon DL. Pharmacological enhancement of synaptic efficacy, spatial learning, and memory through carbonic anhydrase activation in rats. J Pharmacol Exp Ther. 2001;297:961–967.
50. Huang X, Cuajungco MP, Atwood CS, Moir RD, Tanzi RE, Bush AI. Alzheimer's disease, beta-amyloid protein and zinc. J Nutr. 2000;130:1488S-92S.
51. Supuran CT. Carbonic anhydrase activators. Future Med Chem. 2018;10:561–573.
52. Provensi G, Carta F, Nocentini A, Supuran CT, Casamenti F, Passani MB, et al. A New Kid on the Block? Carbonic Anhydrases as Possible New Targets in Alzheimer's Disease. Int J Mol Sci. 2019;20.
53. Canto de Souza L, Provensi G, Vullo D, Carta F, Scozzafava A, Costa A, et al. Carbonic anhydrase activation enhances object recognition memory in mice through phosphorylation of the extracellular signal-regulated kinase in the cortex and the hippocampus. Neuropharmacology. 2017;118:148–156.
54. Thompson PJ, Baxendale SA, Duncan JS, Sander JW. Effects of topiramate on cognitive function. J Neurol Neurosurg Psychiatry. 2000;69:636–641.
55. McPhee I, Cochran S, Houslay MD. The novel long PDE4A10 cyclic AMP phosphodiesterase shows a pattern of expression within brain that is distinct from the long PDE4A5 and short PDE4A1 isoforms. Cell Signal. 2001;13:911–918.
56. Pérez-Torres S, Miró X, Palacios JM, Cortés R, Puigdoménech P, Mengod G. Phosphodiesterase type 4 isozymes expression in human brain examined by in situ hybridization histochemistry and[3H]rolipram binding autoradiography. Comparison with monkey and rat brain. J Chem Neuroanat. 2000;20:349–374.
57. Li Y-F, Huang Y, Amsdell SL, Xiao L, O'Donnell JM, Zhang H-T. Antidepressant- and anxiolytic-like effects of the phosphodiesterase-4 inhibitor rolipram on behavior depend on cyclic AMP response element binding protein-mediated neurogenesis in the hippocampus. Neuropsychopharmacology. 2009;34:2404–2419.
58. Zhang H-T, Zhao Y, Huang Y, Deng C, Hopper AT, De Vivo M, et al. Antidepressant-like effects of PDE4 inhibitors mediated by the high-affinity rolipram binding state (HARBS) of the phosphodiesterase-4 enzyme (PDE4) in rats. Psychopharmacology (Berl). 2006;186:209–217.

59. Block F, Schmidt W, Nolden-Koch M, Schwarz M. Rolipram reduces excitotoxic neuronal damage. Neuroreport. 2001;12:1507–1511.
60. Barad M, Bourtchouladze R, Winder DG, Golan H, Kandel E. Rolipram, a type IV-specific phosphodiesterase inhibitor, facilitates the establishment of long-lasting long-term potentiation and improves memory. Proc Natl Acad Sci USA. 1998;95:15020–15025.
61. Burgin AB, Magnusson OT, Singh J, Witte P, Staker BL, Bjornsson JM, et al. Design of phosphodiesterase 4D (PDE4D) allosteric modulators for enhancing cognition with improved safety. Nat Biotechnol. 2010;28:63–70.
62. Blokland A, Menniti FS, Prickaerts J. PDE inhibition and cognition enhancement. Expert Opin Ther Pat. 2012;22:349–354.
63. Gurney ME. Genetic Association of Phosphodiesterases With Human Cognitive Performance. Front Mol Neurosci. 2019;12:22.
64. Somaini G, Stamm A, Müller-Mottet S, Hasler E, Keusch S, Hildenbrand FF, et al. Disease-Targeted Treatment Improves Cognitive Function in Patients with Precapillary Pulmonary Hypertension. Respiration. 2015;90:376–383.
65. Ende G. Proton Magnetic Resonance Spectroscopy: Relevance of Glutamate and GABA to Neuropsychology. Neuropsychol Rev. 2015;25:315–325.
66. Schmidt-Wilcke T, Fuchs E, Funke K, Vlachos A, Müller-Dahlhaus F, Puts N a. J, et al. GABA-from Inhibition to Cognition: Emerging Concepts. Neuroscientist. 2018;24:501–515.
67. Contractor A, Heinemann SF. Glutamate receptor trafficking in synaptic plasticity. Sci STKE. 2002;2002:re14.
68. Watkins JC, Jane DE. The glutamate story. Br J Pharmacol. 2006;147 Suppl 1:S100-108.
69. Brassai A, Suvanjeiev R-G, Bán E-G, Lakatos M. Role of synaptic and nonsynaptic glutamate receptors in ischaemia induced neurotoxicity. Brain Res Bull. 2015;112:1–6.
70. Mehta A, Prabhakar M, Kumar P, Deshmukh R, Sharma PL. Excitotoxicity: bridge to various triggers in neurodegenerative disorders. Eur J Pharmacol. 2013;698:6–18.
71. Morrow JA, Maclean JKF, Jamieson C. Recent advances in positive allosteric modulators of the AMPA receptor. Curr Opin Drug Discov Devel. 2006;9:571–579.
72. Arai AC, Kessler M. Pharmacology of ampakine modulators: from AMPA receptors to synapses and behavior. Curr Drug Targets. 2007;8:583–602.
73. O’Neill MJ, Dix S. AMPA receptor potentiators as cognitive enhancers. IDrugs. 2007;10:185–192.
74. Cleva RM, Gass JT, Widholm JJ, Olive MF. Glutamatergic targets for enhancing extinction learning in drug addiction. Curr Neuropharmacol. 2010;8:394–408.
75. Lynch G, Palmer LC, Gall CM. The likelihood of cognitive enhancement. Pharmacol Biochem Behav. 2011;99:116–129.
76. Xu M-Y, Wong AHC. GABAergic inhibitory neurons as therapeutic targets for cognitive impairment in schizophrenia. Acta Pharmacol Sin. 2018;39:733–753.
77. Fang Y, Qiu Z, Hu W, Yang J, Yi X, Huang L, et al. Effect of piracetam on the cognitive performance of patients undergoing coronary bypass surgery: A meta-analysis. Exp Ther Med. 2014;7:429–434.
78. Malykh AG, Sadaie MR. Piracetam and piracetam-like drugs: from basic science to novel clinical applications to CNS disorders. Drugs. 2010;70:287–312.
79. Su T, Lu Y, Geng Y, Lu W, Chen Y. How could N-Methyl-D-Aspartate Receptor Antagonists Lead to Excitation Instead of Inhibition? Brain Science Advances. 2018;4:73–98.
80. Olivares D, Deshpande VK, Shi Y, Lahiri DK, Greig NH, Rogers JT, et al. N-methyl D-aspartate (NMDA) receptor antagonists and memantine treatment for Alzheimer’s disease, vascular dementia and Parkinson’s disease. Curr Alzheimer Res. 2012;9:746–758.
81. Imbrici P, Camerino DC, Tricarico D. Major channels involved in neuropsychiatric disorders and therapeutic perspectives. Front Genet. 2013;4.
82. Noebels J. Precision physiology and rescue of brain ion channel disorders. J Gen Physiol. 2017;149:533–546.

83. Clapham DE. Calcium signaling. Cell. 2007;131:1047–1058.
84. Biala G, Kruk-Slomka M, Jozwiak K. Influence of acute or chronic calcium channel antagonists on the acquisition and consolidation of memory and nicotine-induced cognitive effects in mice. Naunyn Schmiedebergs Arch Pharmacol. 2013;386:651–664.
85. Chen C-C, Shen J-W, Chung N-C, Min M-Y, Cheng S-J, Liu IY. Retrieval of context-associated memory is dependent on the Ca(v)3.2 T-type calcium channel. PLoS ONE. 2012;7:e29384.
86. Deyo RA, Hittner JM. Effects of the Ca2+ channel antagonist flunarizine on visual discrimination learning. Neurobiol Learn Mem. 1995;64:10–16.
87. Disterhoft JF, Moyer JR, Thompson LT. The calcium rationale in aging and Alzheimer's disease. Evidence from an animal model of normal aging. Ann N Y Acad Sci. 1994;747:382–406.
88. Foster TC. Dissecting the age-related decline on spatial learning and memory tasks in rodent models: N-methyl-D-aspartate receptors and voltage-dependent Ca2+ channels in senescent synaptic plasticity. Prog Neurobiol. 2012;96:283–303.
89. Hopp SC, D'Angelo HM, Royer SE, Kaercher RM, Crockett AM, Adzovic L, et al. Calcium dysregulation via L-type voltage-dependent calcium channels and ryanodine receptors underlies memory deficits and synaptic dysfunction during chronic neuroinflammation. J Neuroinflammation. 2015;12:56.
90. Marschallinger J, Sah A, Schmuckermair C, Unger M, Rotheneichner P, Kharitonova M, et al. The L-type calcium channel Cav1.3 is required for proper hippocampal neurogenesis and cognitive functions. Cell Calcium. 2015;58:606–616.
91. Yan QS, Reith ME, Jobe PC, Dailey JW. Dizocilpine (MK-801) increases not only dopamine but also serotonin and norepinephrine transmissions in the nucleus accumbens as measured by microdialysis in freely moving rats. Brain Res. 1997;765:149–158.
92. Dragicevic E, Schiemann J, Liss B. Dopamine midbrain neurons in health and Parkinson's disease: emerging roles of voltage-gated calcium channels and ATP-sensitive potassium channels. Neuroscience. 2015;284:798–814.
93. Etou K, Kuroki T, Tatebayashi Y, Tashiro N, Hirano M. Effects of calcium antagonists nifedipine and flunarizine on phencyclidine-induced changes in the regional dopaminergic metabolism of the rat brain. Biochem Pharmacol. 1996;51:83–86.
94. Anekonda TS, Quinn JF. Calcium channel blocking as a therapeutic strategy for Alzheimer's disease: the case for isradipine. Biochim Biophys Acta. 2011;1812:1584–1590.
95. Krajnak K, Dahl R. A new target for Alzheimer's disease: A small molecule SERCA activator is neuroprotective in vitro and improves memory and cognition in APP/PS1 mice. Bioorg Med Chem Lett. 2018;28:1591–1594.
96. Britzolaki A, Saurine J, Flaherty E, Thelen C, Pitychoutis PM. The SERCA2: A Gatekeeper of Neuronal Calcium Homeostasis in the Brain. Cell Mol Neurobiol. 2018;38:981–994.
97. Rinke I, Artmann J, Stein V. ClC-2 voltage-gated channels constitute part of the background conductance and assist chloride extrusion. J Neurosci. 2010;30:4776–4786.
98. van der Knaap MS, Depienne C, Sedel F, Abbink TE. CLCN2-Related Leukoencephalopathy. In: Adam MP, Ardinger HH, Pagon RA, Wallace SE, Bean LJ, Stephens K, et al., editors. GeneReviews®, Seattle (WA): University of Washington, Seattle; 1993.
99. Kleefuss-Lie A, Friedl W, Cichon S, Haug K, Warnstedt M, Alekov A, et al. CLCN2 variants in idiopathic generalized epilepsy. Nat Genet. 2009;41:954–955.
100. Niemeyer MI, Cid LP, Sepúlveda FV, Blanz J, Auberson M, Jentsch TJ. No evidence for a role of CLCN2 variants in idiopathic generalized epilepsy. Nat Genet. 2010;42:3.
101. Fernandes-Rosa FL, Daniil G, Orozco IJ, Göppner C, El Zein R, Jain V, et al. A gain-of-function mutation in the CLCN2 chloride channel gene causes primary aldosteronism. Nat Genet. 2018;50:355–361.
102. Gras-Miralles B, Cremonini F. A critical appraisal of lubiprostone in the treatment of chronic constipation in the elderly. Clin Interv Aging. 2013;8:191–200.

103. Al-Badri G, Leggio GM, Musumeci G, Marzagalli R, Drago F, Castorina A. Tackling dipeptidyl peptidase IV in neurological disorders. Neural Regen Res. 2018;13:26–34.
104. Bachor TP, Marquioni-Ramella MD, Suburo AM. Sitagliptin protects proliferation of neural progenitor cells in diabetic mice. Metab Brain Dis. 2015;30:885–893.
105. Röhnert P, Schmidt W, Emmerlich P, Goihl A, Wrenger S, Bank U, et al. Dipeptidyl peptidase IV, aminopeptidase N and DPIV/APN-like proteases in cerebral ischemia. J Neuroinflammation. 2012;9:44.
106. Rizzo MR, Barbieri M, Boccardi V, Angellotti E, Marfella R, Paolisso G. Dipeptidyl peptidase-4 inhibitors have protective effect on cognitive impairment in aged diabetic patients with mild cognitive impairment. J Gerontol A Biol Sci Med Sci. 2014;69:1122–1131.
107. Isik AT, Soysal P, Yay A, Usarel C. The effects of sitagliptin, a DPP-4 inhibitor, on cognitive functions in elderly diabetic patients with or without Alzheimer's disease. Diabetes Res Clin Pract. 2017;123:192–198.
108. Darsalia V, Johansen OE, Lietzau G, Nyström T, Klein T, Patrone C. Dipeptidyl Peptidase-4 Inhibitors for the Potential Treatment of Brain Disorders; A Mini-Review With Special Focus on Linagliptin and Stroke. Front Neurol. 2019;10.
109. Kosaraju J, Holsinger RMD, Guo L, Tam KY. Linagliptin, a Dipeptidyl Peptidase-4 Inhibitor, Mitigates Cognitive Deficits and Pathology in the 3xTg-AD Mouse Model of Alzheimer's Disease. Mol Neurobiol. 2017;54:6074–6084.
110. Dong Q, Teng S-W, Wang Y, Qin F, Li Y, Ai L-L, et al. Sitagliptin protects the cognition function of the Alzheimer's disease mice through activating glucagon-like peptide-1 and BDNF-TrkB signalings. Neurosci Lett. 2019;696:184–190.
111. Remaud S, Gothié J-D, Morvan-Dubois G, Demeneix BA. Thyroid hormone signaling and adult neurogenesis in mammals. Front Endocrinol (Lausanne). 2014;5:62.
112. de Escobar GM, Obregón MJ, del Rey FE. Iodine deficiency and brain development in the first half of pregnancy. Public Health Nutr. 2007;10:1554–1570.
113. de Escobar GM, Obregón MJ, del Rey FE. Maternal thyroid hormones early in pregnancy and fetal brain development. Best Pract Res Clin Endocrinol Metab. 2004;18:225–248.
114. Smith JW, Evans AT, Costall B, Smythe JW. Thyroid hormones, brain function and cognition: a brief review. Neurosci Biobehav Rev. 2002;26:45–60.
115. Bauer M, Heinz A, Whybrow PC. Thyroid hormones, serotonin and mood: of synergy and significance in the adult brain. Mol Psychiatry. 2002;7:140–156.
116. Henley WN, Koehnle TJ. Thyroid hormones and the treatment of depression: an examination of basic hormonal actions in the mature mammalian brain. Synapse. 1997;27:36–44.
117. Fu AL, Zhou CY, Chen X. Thyroid hormone prevents cognitive deficit in a mouse model of Alzheimer's disease. Neuropharmacology. 2010;58:722–729.
118. Kramer CK, von Mühlen D, Kritz-Silverstein D, Barrett-Connor E. Treated hypothyroidism, cognitive function, and depressed mood in old age: the Rancho Bernardo Study. Eur J Endocrinol. 2009;161:917–921.
119. Rivas M, Naranjo JR. Thyroid hormones, learning and memory. Genes Brain Behav. 2007;6 Suppl 1:40–44.
